# A CRISPR–Cas9 gene drive targeting *doublesex* causes complete population suppression in caged *Anopheles gambiae* mosquitoes

**DOI:** 10.1038/nbt.4245

**Published:** 2018-09-24

**Authors:** Kyros Kyrou, Andrew M Hammond, Roberto Galizi, Nace Kranjc, Austin Burt, Andrea K Beaghton, Tony Nolan, Andrea Crisanti

**Affiliations:** Department of Life Sciences, Imperial College London, UK

**Keywords:** Genetic engineering, Genetic techniques

## Abstract

In the human malaria vector *Anopheles gambiae*, the gene *doublesex* (*Agdsx*) encodes two alternatively spliced transcripts, *dsx-female* (*AgdsxF*) and *dsx-male* (*AgdsxM*), that control differentiation of the two sexes. The female transcript, unlike the male, contains an exon (exon 5) whose sequence is highly conserved in all *Anopheles* mosquitoes so far analyzed. We found that CRISPR–Cas9-targeted disruption of the intron 4–exon 5 boundary aimed at blocking the formation of functional AgdsxF did not affect male development or fertility, whereas females homozygous for the disrupted allele showed an intersex phenotype and complete sterility. A CRISPR–Cas9 gene drive construct targeting this same sequence spread rapidly in caged mosquitoes, reaching 100% prevalence within 7–11 generations while progressively reducing egg production to the point of total population collapse. Owing to functional constraint of the target sequence, no selection of alleles resistant to the gene drive occurred in these laboratory experiments. Cas9-resistant variants arose in each generation at the target site but did not block the spread of the drive.

**Supplementary information:**

The online version of this article (doi:10.1038/nbt.4245) contains supplementary material, which is available to authorized users.

## Abstract (web summary)

Complete population collapse of malaria vector *Anopheles gambiae* in cages is achieved using a gene drive that targets *doublesex*.

**Supplementary information:**

The online version of this article (doi:10.1038/nbt.4245) contains supplementary material, which is available to authorized users.

## Main

CRISPR–Cas9 nucleases have been applied in gene drive constructs to target endogenous sequences of the human malaria vectors*A. gambiae* and *A. stephensi* with the objective of vector control^1,2^. These proof-of-principle experiments translated a hypothesis into a genetic tool able to suppress the reproductive capability of the mosquito population. According to mathematical modeling, suppression of *A. gambiae* mosquito reproductive capability can be achieved using gene drive systems targeting haplosufficient female fertility genes^3,4^ or by introducing a sex distorter on the Y chromosome in the form of a nuclease designed to shred the X chromosome during meiosis, an approach known as Y-drive^4,5,6^. Both strategies could cause a progressive decrease in the number of fertile females that would eventually collapse the population.

However, several technical and scientific issues remain before these proof-of-principle demonstrations are advanced to effect vector population suppression. The development of a Y-drive has so far proven difficult because of the complete transcriptional shut down of the sex chromosomes during meiosis, which prevents the expression of a Y-linked sex distorter during gamete formation^6,7^. A gene drive designed to disrupt the *A. gambiae* fertility gene *AGAP007280* initially increased in frequency, but the selection of nuclease-resistant, functional variants that could be detected as early as generation 2 completely blocked the spread of the drive^2^. Resistant variants comprised small insertions or deletions (indels) of differing length generated by nonhomologous end joining repair following nuclease activity at the target site. The development of resistance to any nuclease-based gene drive was predicted^3^ and is regarded as the main technical obstacle for the use of gene drives for vector control^8,9,10,11,12^ (Supplementary Table 1). Gene drive targets with functional or structural constraints that might prevent the development of resistant variants could offer a route to successful population control. With this in mind, we evaluated the potential for disruption of the sex determination pathway in *A. gambiae* mosquitoes to selectively block the formation of the female splice transcript of the gene *doublesex* (*dsx*).

## Results

### *doublesex* and sex differentiation in *A. gambiae*

**Figure 1:**
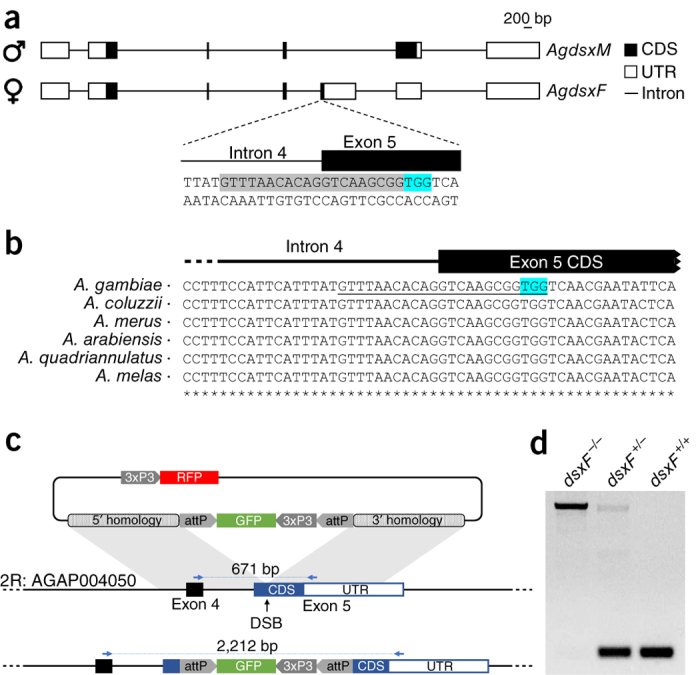
Targeting the female-specific isoform of *doublesex*. (**a**) Schematic representation of the male- and female-specific *dsx* transcripts and the gRNA sequence used to target the gene (shaded in gray). The gRNA spans the intron 4–exon 5 boundary. The protospacer-adjacent motif (PAM) of the gRNA is highlighted in blue. Scale bar, 200 bp. Coding regions of exons (CDS) are shaded in black, noncoding regions in white. Introns are not drawn to scale. UTR, untranslated region. (**b**) Sequence alignment of the *dsx* intron 4–exon 5 boundary in six of the species from the *A. gambiae* complex. The sequence is highly conserved within the complex suggesting tight functional constraint at this region of the *dsx* gene. The gRNA used to target the gene is underlined and the protospacer-adjacent motif is highlighted in blue. (**c**) Schematic representation of the HDR knockout construct specifically recognizing exon 5 and the corresponding target locus. DSB, double-strand break. (**d**) Diagnostic PCR using a primer set (blue arrows in **c**) to discriminate between the wild-type and *dsxF* allele in homozygous (*dsxF*^*−/−*^), heterozygous (*dsxF*^*+/−*^) and wild-type (*dsxF*^+/+^) individuals.

Sex differentiation in insects follows a common pattern in which a primary signal activates a central gene that induces a cascade of molecular mechanisms that control alternative splicing of the *doublesex* (*dsx*) gene^13,14^. Although the molecular mechanisms and the genes involved in regulating sex differentiation in *A. gambiae* are not well understood, except that *Yob1* functions as a Y-linked male determining factor^15^, available data indicated an important role of *dsx* in determining sexual dimorphism in this mosquito species^16^. In *A. gambiae*, *dsx* (*Agdsx*) consists of seven exons, distributed over an 85-kb region on chromosome 2R, a gene structure similar to that of *Drosophila melanogaster dsx* (*Dmdsx*) and other insect orthologs, and is alternately spliced to produce the female and male transcripts *AgdsxF* and *AgdsxM*, respectively. The female transcript consists of a 5′ segment common with that of males, a highly conserved female-specific exon (exon 5) and a 3′ common region, while the male transcript comprises only the 5′ and 3′ common segments. The male-specific isoform contains an additional domain at the C terminus that is transcribed as a noncoding 3′ untranslated region in females (Fig. 1a).

**Figure 2:**
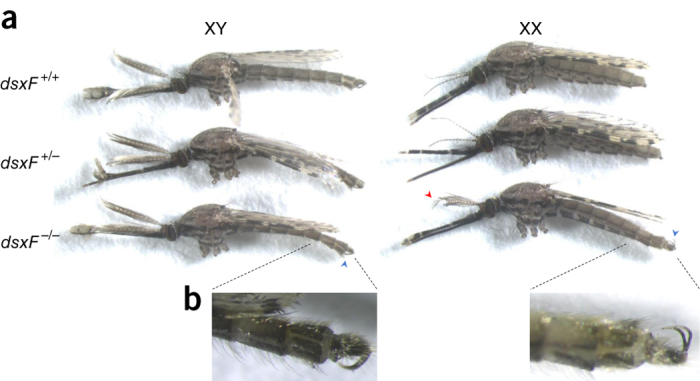
Morphological analysis of homozygous *dsxF*^*−/−*^ mutants. (**a**) Morphological appearance of genetic males and females heterozygous (*dsxF*^*+/−*^) or homozygous (*dsxF*^*−/−*^) for the exon 5 null allele. This assay was performed in a strain containing a dominant RFP marker linked to the Y chromosome, whose presence permits unambiguous determination of male or female genotype. Anomalies in sexual morphology were observed only in *dsxF*^*−/−*^ genetic female mosquitoes. This group of XX individuals showed male-specific traits, including a plumose antenna (red arrowhead) and claspers (blue arrowheads). This group also showed anomalies in the proboscis and accordingly they could not bite and feed on blood. Representative samples of each genotype are shown. (**b**) Magnification of the external genitalia. All *dsxF*^*−/−*^ females carried claspers, a male-specific characteristic. The claspers were dorsally rotated rather than in the normal ventral position.

**Figure 3:**
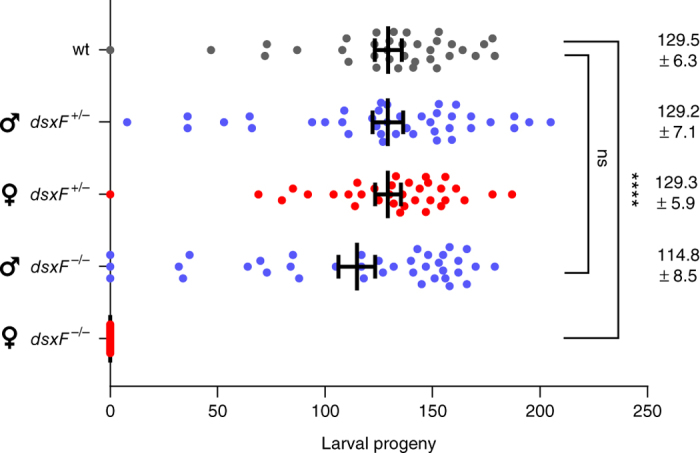
Reproductive phenotype of *dsxF* mutants. Male and female *dsxF*^*−/−*^ and *dsxF*^*+/−*^ individuals were mated with the corresponding wild-type sexes. Females were given access to a blood meal and subsequently allowed to lay individually. Fecundity was investigated by counting the number of larval progeny per lay (*n* ≥ 43). Using wild type (wt) as a comparator, we saw no significant differences ('ns') in any genotype other than *dsxF*^*−/−*^ females, which were unable to feed on blood and therefore failed to produce a single egg (****P < 0.0001; Kruskal–Wallis test). Vertical bars indicate the mean and the s.e.m. Blue and red indicate the crosses of male or female *dsxF* mutants, respectively, to wild type, whereas the gray dots represent wild-type-only crosses.

To investigate whether *dsx* is a suitable target for a gene drive to suppress population reproductive capacity, we disrupted the intron 4–exon 5 boundary of *dsx* (Fig. 1b) to prevent the formation of functional AgdsxF while leaving the AgdsxM transcript unaffected. We injected *A. gambiae* embryos with a source of Cas9 and a single-guide RNA (sgRNA) designed to recognize and cleave a sequence overlapping the intron 4–exon 5 boundary, in combination with a template for homology-directed repair (HDR) to insert an eGFP transcription unit (Fig. 1c). Transformed individuals were intercrossed to generate homozygous and heterozygous mutants among the progeny. HDR-mediated integration was confirmed with diagnostic PCR using primers that spanned the insertion site: a large amplicon for the HDR event and a smaller amplicon for the wild-type allele enabled facile confirmation of genotypes (Fig. 1d). The knock-in of eGFP resulted in the complete disruption of the exon 5 (*dsxF*^*−*^) coding sequence and was confirmed by PCR and genomic sequencing of the chromosomal integration (Supplementary Fig. 1). Crosses of heterozygous individuals produced wild-type, heterozygous and homozygous individuals for the *dsxF*^−^ allele at the expected Mendelian ratio 1:2:1, indicating that there was no obvious lethality associated with the mutation during development (Supplementary Table 2). Larvae heterozygous for the exon 5 disruption developed into adult male and female mosquitoes with a sex ratio close to 1:1. However, half of *dsxF*^*−/−*^ individuals developed into normal males whereas the other half had both male and female morphological features, as well as a number of developmental anomalies in the internal and external reproductive organs (intersex phenotype). To establish the sex genotype of these *dsxF*^*−/−*^ intersex mosquitoes, we introgressed the mutation into a line containing a Y-linked visible marker (RFP) and used the presence of this marker to unambiguously assign sex genotype among individuals heterozygous and homozygous for the null mutation. This approach revealed that the intersex phenotype was observed only in females that were homozygous for the null mutation. We saw no phenotype in heterozygous mutants, suggesting that the female-specific isoform of *dsx* is haplosufficient. Examination of external sexually dimorphic structures in *dsxF*^*−/−*^ genotypic females (*n* > 50) showed several phenotypic abnormalities, including the development of dorsally rotated male claspers (and absence of female cerci) and longer flagellomeres associated with male-like plumose antennae (Fig. 2 and Supplementary Table 3). Analyses of the internal reproductive organs of the same set of insects revealed the absence of fully developed ovaries and spermathecae; instead these were replaced with male accessory glands and in some cases (∼20%) by rudimentary pear-shaped organs resembling unstructured testes (Supplementary Fig. 2). Males carrying the *dsxF*^*−*^ null mutation in heterozygosity or homozygosity showed wild-type levels of fertility as measured by clutch size and larval hatching per mated female, as did heterozygous *dsxF*^*−*^ female mosquitoes. Intersex XX *dsxF*^*−/−*^ female mosquitoes, although attracted to anesthetized mice, were unable to take a blood meal and failed to produce any eggs (Fig. 3). The drastic phenotype of *dsxF*^*−/−*^ in females indicates that exon 5 of *dsx* has a fundamental role in the previously poorly understood sex differentiation pathway of *A. gambiae* mosquitoes and suggested that its sequence might represent a suitable target for gene drives designed for population suppression.

### Building a gene drive to target *dsx*

**Figure 4:**
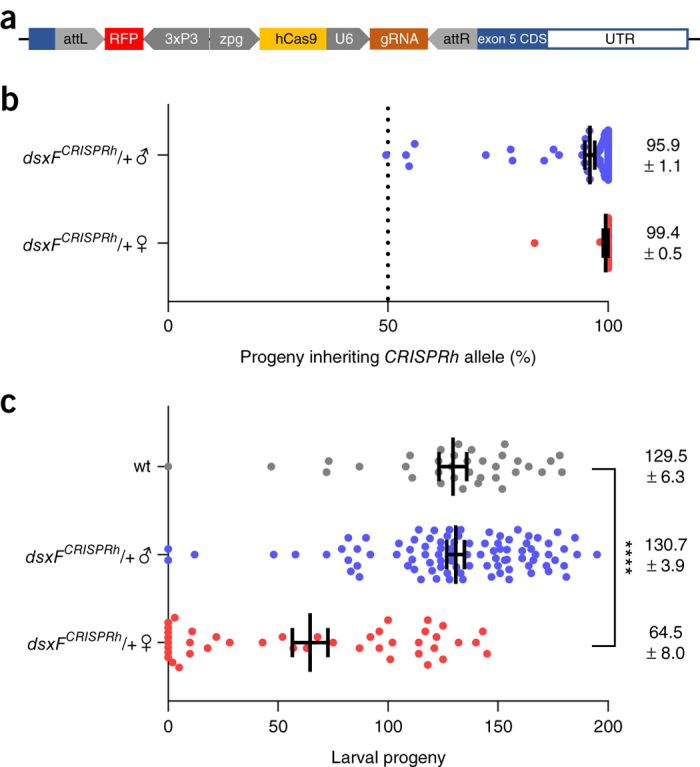
Transmission rate of the *dsxF*^*CRISPRh*^ driving allele and fecundity analysis of heterozygous male and female mosquitoes. (**a**–**c**) Male and female mosquitoes heterozygous for the *dsxF*^*CRISPRh*^ allele (**a**) were analyzed in crosses with wild-type mosquitoes to assess the inheritance bias of the *dsxF*^*CRISPRh*^ drive construct (**b**) and for the effect of the construct on their reproductive phenotype (**c**). (**b**) Scatter plot of the transgenic rate observed in the progeny of *dsxF*^*CRISPRh*^/+ female or male mosquitoes that gave progeny when crossed to wild-type individuals (*n* ≥ 33). Each dot represents the progeny derived from a single female. Both male and female *dsxF*^*CRISPRh*^/+ showed a high transmission rate of up to 100% of the *dsxF*^*CRISPRh*^ allele to the progeny. The transmission rate was determined by visually scoring offspring for the RFP marker that is linked to the *dsxF*^*CRISPRh*^ allele. The dotted line indicates the expected Mendelian inheritance. Mean transmission rate (± s.e.m.) is shown. (**c**) Scatter plot showing the number of larvae produced by single females (*n* ≥ 35) from crosses of *dsxF*^*CRISPRh*^/+ mosquitoes with wild-type individuals after one blood meal. Mean progeny count (± s.e.m.) is shown (****P < 0.0001; Kruskal–Wallis test).

We used recombinase-mediated cassette exchange to replace the *3xP3::GFP* transcription unit with a *dsxF*^*CRISPRh*^ gene drive construct that comprised an RFP marker gene, a transcription unit to express the guide RNA (gRNA) targeting *dsxF*, and *cas9* under the control of the germline promoter of *zero population growth* (*zpg*) and its terminator sequence (Fig. 4a and Supplementary Fig. 3). The *zpg* promoter has improved germline restriction of expression, resulting in increased female fertility compared with the *vasa* promoter used in previous gene drive constructs^17^ (Supplementary Fig. 4). Successful cassette exchange events that incorporated *dsxF*^*CRISPRh*^ into the target locus were confirmed in those individuals that had swapped the GFP for the RFP marker (*n* = 17 G_1_ individuals) (Supplementary Fig. 3). During meiosis the Cas9–gRNA complex cleaves the wild-type allele at the target sequence and the *dsxF*^*CRISPRh*^ cassette is copied into the wild-type locus by HDR ('homing'), disrupting exon 5 in the process. The ability of the *dsxF*^*CRISPRh*^ construct to home and bypass Mendelian inheritance was analyzed by scoring the rates of RFP inheritance in the progeny of heterozygous parents (referred to as *dsxF*^*CRISPRh*^/+ hereafter) crossed to wild-type mosquitoes. High *dsxF*^*CRISPRh*^ transmission rates were observed in the progeny of both heterozygous *dsxF*^*CRISPRh*^/+ male (95.9% ± 1.1% s.e.m.; *n* = 87) and female mosquitoes (99.4% ± 0.5%; *n* = 33) (Fig. 4b). The fertility of the *dsxF*^*CRISPRh*^ line was also assessed to unravel potential negative effects due to ectopic expression of the nuclease in somatic cells and/or parental deposition of the nuclease into the newly fertilized embryos (Fig. 4c). These experiments showed that while heterozygous *dsxF*^*CRISPRh*^/+ males showed a fecundity rate (assessed as larval progeny per fertilized female) that did not differ from that of wild-type males, heterozygous *dsxF*^*CRISPRh*^/+ females had reduced fecundity overall (mean fecundity 49.8% ± 6.3% s.e.m., *P* < 0.0001). We noticed a greater reduction in the fertility of heterozygous females when the drive allele was inherited from the father (mean fecundity 21.7% ± 8.6%; *P* < 0.0001) (*n* = 15) rather than the mother (64.9% ± 6.9%; *P* < 0.001) (*n* = 28) (Supplementary Fig. 5). This could be explained by assuming a paternal deposition of active Cas9 nuclease into the newly fertilized zygote that stochastically induces conversion of *dsx* to *dsxF*^*−*^, either through end-joining or HDR, in a substantial number of embryonic cells, which in females results in a reduced fertility. Consistent with this hypothesis, some heterozygous females (9 of 31 examined) receiving a paternal *dsxF*^*CRISPRh*^ allele showed a somatic mosaic phenotype that included, with varying penetrance, the absence of spermatheca and/or the formation of an incomplete clasper set (Supplementary Fig. 2c).

### Assessment of *dsx* gene drive in caged insects

**Figure 5:**
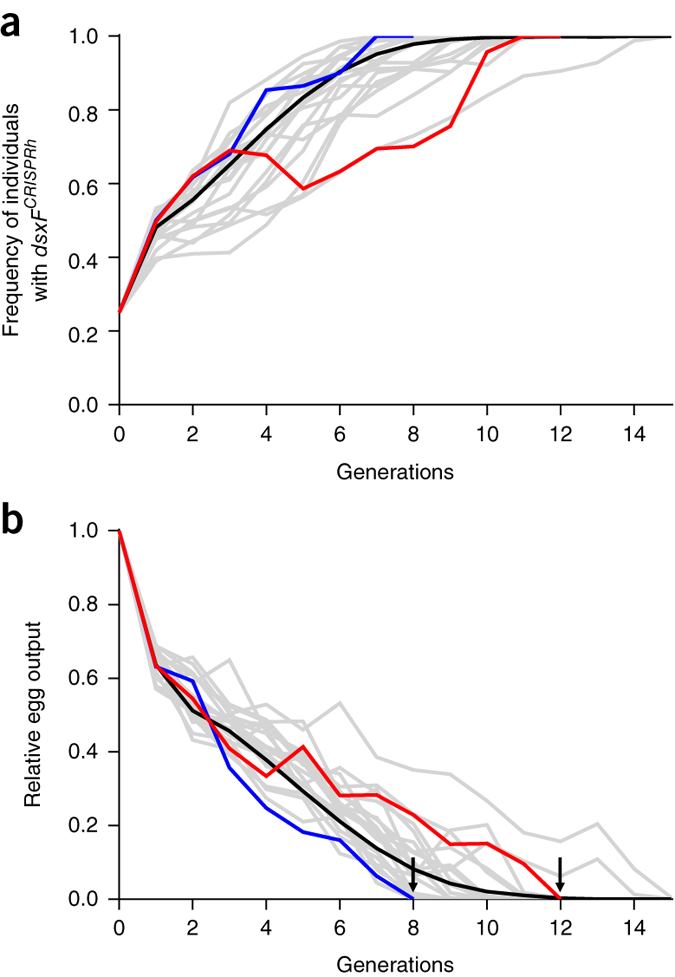
Dynamics of the spread of the *dsxF*^*CRISPRh*^ allele and effect on population reproductive capacity. Two cages were set up with a starting population of 300 wild-type females, 150 wild-type males and 150 *dsxF*^*CRISPRh*^/+ males, seeding each cage with a *dsxF*^*CRISPRh*^ allele frequency of 12.5%. (**a**) The frequency of *dsxF*^*CRISPRh*^ mosquitoes was scored for each generation. The drive allele reached 100% prevalence in both cage 2 (blue) and cage 1 (red) at generation 7 and 11, respectively, in agreement with a deterministic model (black line) that takes into account the parameter values retrieved from the fecundity assays. Twenty stochastic simulations were run (gray lines) assuming a maximum population size of 650 individuals. (**b**) Total egg output deriving from each generation of the cage was measured and normalized relative to the output from the starting generation. Suppression of the reproductive output of each cage led the population to collapse completely (black arrows) by generation 8 (cage 2) or generation 12 (cage 1). Parameter estimates included in the model are provided in Supplementary Table 5.

Using a mathematical model that includes the inheritance bias of the construct, the fecundity of heterozygous individuals, the phenotype of intersex, and the effect of the paternal deposition of the nuclease on female fertility (Online Methods), we found that the *dsxF*^*CRISPRh*^ had the potential to reach 100% frequency in caged population in 9–13 generations considering a starting allele frequency of 12.5% and stochasticity (Fig. 5a). To test this hypothesis, we mixed caged wild-type mosquito populations with heterozygous individuals carrying the *dsxF*^*CRISPRh*^ allele and monitored progeny at each generation to assess the spread of the drive and to quantify effect(s) on reproductive output. We started the experiment in two replicate cages, each with an initial drive allele frequency of 12.5% (300 wild-type female mosquitoes with 150 wild-type male mosquitoes and 150 *dsxF*^*CRISPRh*^/+ male individuals). The initial drive allele frequency that we selected minimizes the stochastic loss of the drive (Supplementary Fig. 6) and represents a realistic field release scenario, being severalfold lower than that used in non-invasive genetic control strategies^18^. All of the eggs produced by the entire cage population were counted, and then 650 eggs were randomly selected to seed the next generations. The larvae that hatched from the eggs were counted and screened for the presence of the RFP marker to score the number of the progeny containing the *dsxF*^*CRISPRh*^ allele in each generation.

During the first three generations we observed an increase of the drive allele from 25% to ∼69% in both caged populations, but at generation 4 the outcomes in the two cages diverged. In cage 2 the drive reached 100% frequency by generation 7; in generation 8, no eggs were produced and the population collapsed. In cage 1 the drive allele reached 100% frequency at generation 11 after remaining at around 65–70% for generations 4 through 8. In generation 12 the cage 1 population also failed to produce eggs (Fig. 5b). While the dynamics of spread of the gene drive in the two caged populations was different, both sets of finding fall within the prediction range of our mathematical model (Fig. 5).

### Potential for resistance to *dsx* gene drive

We monitored the occurrence of mutations at the drive target site in generations 2, 3, 4 and 5 to identify the occurrence of nuclease-resistant, functional variants. Amplicon sequencing of the target sequence from pooled samples containing a minimum of 359 mosquitoes, which were collected in generations 2–5, revealed several low-frequency indels present at the target site (up to 1.16% frequency among nondrive alleles), none of which appeared to encode a functional *AgdsxF* transcript (Supplementary Fig. 7). In addition, none of the variants identified showed any signs of positive selection, which would be expected to cause them to increase in frequency as the drive progressively increased in frequency over generations, suggesting that the selected target sequence has rigid functional or structural constraints. This hypothesis is supported by the exceptionally high conservation of exon 5 in *A. gambiae* mosquitoes^19,20^ and the presence of a strictly regulated splice site that is crucial in mosquito reproductive biology. Furthermore, large-scale resequencing of 765 wild-caught mosquitoes from eight sub-Saharan African countries^20^ revealed only a single rare SNP within the drive target site, present at 2.9% frequency (Supplementary Fig. 8). This naturally occurring variant could block the spread of the drive. To investigate this hypothesis, we tested whether this SNP variant was as susceptible to cleavage *in vitro* by Cas9 as the wild-type sequence, using the sgRNA from our gene drive construct. We found that the gRNA in our gene drive construct efficiently cleaved both the wild-type and the SNP sequence variant, which may indicate that our gene drive would be able to spread even if this conserved SNP was present (Supplementary Fig. 9). However, it is important to note that we cannot state that our drive target site is 'resistance-proof', since at scale, and over time, it is possible that nuclease-induced mutations could be produced that do restore sufficient function to the gene to be positively selected. This notwithstanding, targeting gene drives to functionally constrained sequences is clearly advantageous, as evidenced by the population collapse effected by this gene drive in both caged mosquito populations. Distinct, highly conserved sequences may have varying levels of functional constraint, and the relative strength of selection for maintaining sequence conservation versus the strength of selection imposed by the gene drive will ultimately determine their suitability as targets for gene drives.

Our data not only provide important functional insights into the role of *dsx* in *A. gambiae* sex determination, but also represent a substantial step toward the development of effective gene drive vector-control measures that aim to suppress insect populations. The intersex phenotype of *dsxF*^*−/−*^ genetic females shows that exon 5 is crucial for the production of a functional female transcript, as was initially hypothesized on the basis of the expression profile of the *dsx* splice variants in the two sexes^16^. Furthermore, the observation that heterozygous *dsxF*^*CRISPRh*^/+ females are fertile and produce almost 100% inheritance of the drive might indicate that most of the germ cells in these females are homozygous and, unlike somatic cells, do not undergo autonomous *dsx*-mediated sex commitment^21^.

## Discussion

The development of a gene drive capable of collapsing a human malaria vector population to levels that cannot support malaria transmission is a long-sought scientific and technical goal^22^. The gene drive *dsxF*^*CRISPRh*^ targeting exon 5 of *dsx* has several features that make it suitable for future field testing. Specifically, this drive has high inheritance bias, heterozygous individuals are fully fertile, homozygous females are sterile and unable to bite, and we found no evidence for nuclease-resistant functional variants at the drive target site. We note that these proof-of-principle experiments cannot conclude that this drive is resistance proof. This is in contrast to a recent study in *Drosophila* that targeted the *transformer* gene, upstream of *doublesex*. Invasion of the drive in *transformer* was rapidly compromised by the accumulation of large numbers of functional and nonfunctional resistant alleles^23^.

Our *doublesex* gene drive now needs to be rigorously evaluated in large confined spaces that more closely mimic native ecological conditions, in accordance with the recommendations of the US National Academy of Sciences^24^. Under such conditions, competition for resources or mating success may disproportionately affect individuals harboring the gene drive, resulting in invasion dynamics substantially different from those observed in insectary cage experiments. Indeed, previous work with other genetically manipulated insects would suggest that in the less ideal conditions present in field cages and natural landscapes (competition for food, presence of predators and environmental stressors), heterozygous female mosquitoes carrying the drive allele might have a further reduction in fitness as result of the combined effect of the genetic background of the laboratory strain and the presence of the drive construct itself (Supplementary Table 1)^25,26,27^. To mimic less ideal conditions, we modeled varying levels of additional reduction in fitness (over the experimentally observed value of reproduction rate) associated with the heterozygous gene drive and evaluated the effects on penetrance of the *doublesex* gene drive (Supplementary Fig. 10). An additional reduction in fitness (over the experimentally observed value) of up to 40% would still allow the drive to reach 100% frequency and cause population suppression, albeit more slowly. Further reductions in fitness would result in different equilibrium frequencies that might still cause a large reproductive load on the population.

Our results may have implications beyond malaria vector control. The role of *doublesex* in sex determination in all insect species so far analyzed, and the high degree of *doublesex* sequence conservation among members of the same species (in gene regions involved in sex-specific splicing), suggests that these sequences might be an Achilles heel present in many insect pests that could be targeted with gene editing approaches.

## Methods

### Choice of target site.

To selectively disrupt the female-specific isoform of *dsx* we targeted the upstream intron–exon boundary of exon 5, which has been shown to be expressed only in the female mosquito^16^. This exon spans a region of 1,712 bp on chromosome 2R (48,712,937–48,714,648) and contains at its 5′ end 89 bp encoding the sequence-specific portion of the female *A. gambiae dsx* isoform (AgdsxF). We identified a potential gRNA target site that showed almost complete sequence conservation across 16 different anopheline species and complete conservation across the *A. gambiae* species complex^19^ (viewed using http://people.csail.mit.edu/waterhouse/alnloc.cgi), with no nucleotide variation at 22 of the 23 targeted bases across 765 wild-caught *A. gambiae* collected as part of the *Anopheles gambiae* 1000 Genomes project^20^. A single nucleotide variant existing in the target site was represented at 2.9% allele frequency in the wild-caught mosquitoes (Supplementary Fig. 8). *In vitro* testing of this SNP variant revealed it to be as susceptible as the wild-type sequence to Cas9 cleavage directed by the gRNA used in our gene drive construct (Supplementary Fig. 9). The gRNA target and protospacer-adjacent motif (5′-GTTTAACACAGGTCAAGCGGTGG-3′) was also assessed *in silico* for off-target activity using the online-based tool ChopChop (http://chopchop.cbu.uib.no)^28,29^.

### Generation of CRISPR and donor constructs.

We engineered available template plasmids to develop the CRISPR (p16510) and donor (pK101) constructs used to induce a double-strand break on the *dsx* target sequence and to provide template for homology-mediated repair, respectively. In practice a CRISPR construct^2^ containing a U6::gRNA spacer cloning cassette was utilized, using Golden Gate cloning, to generate a PolIII transcription unit containing the *dsx*-specific gRNA. The plasmid also contained a human-codon-optimized *Cas9* coding sequence (*hCas9*) under the control of the *vasa2* promoter, which directs the expression of the Cas9 protein in the pole cells of the developing embryo. The donor plasmid was designed to contain a *GFP* transcription unit under the control of the *3xP3* promoter enclosed within two reversible ϕC31 *attP* recombination sequences flanked both 5′ and 3′ by 2 kb sequence immediately upstream and downstream, respectively, of the target site in *dsx* exon 5. The homology recombination regions flanking the *dsx* target site were amplified using primers adapted for Gibson assembly (dsxϕ31L-F + dsxϕ31L-R, dsxϕ31R-F + dsxϕ31R-R) (Supplementary Table 4), and the 3xP3::GFP cassette and backbone were excised using restriction enzymes from plasmid p163 (ref. 2). The final donor vector was named K101 (GenBank accession code MH541846) and was assembled using the standard Gibson assembly protocol^30^.

### Generation of the *dsxF* CRISPR homing allele (*dsxF*^*CRISPRh*^).

The *dsxF*^*CRISPRh*^ homing allele was generated *in vivo* by ϕC31 recombinase-mediated cassette exchange (RMCE)^31^ using construct p17410, which encompassed the *hCas9* and the *dsx* gRNA transcription units, as well as reporter 3xP3::RFP cassette within two reversible ϕC31 *attB* recombination sequences. The gene drive construct targeting *dsxF* is identical in design to that described in Hammond *et al*.^2^ except for the promoter and 3′ UTR surrounding the Cas9 gene: where previously these were from the ortholog of *vasa* (*AGAP008578*), in the current construct these are replaced by 1,074 bp upstream and 1,034 bp downstream of the germline-specific gene *AGAP006241*, the putative ortholog of *zero population growth* (*zpg*). A comparison of the fertility and homing rates in individuals heterozygous *vasa*- and *zpg*-driven gene CRISPR^h^ constructs at the exact same target locus (in *AGAP007280*, previously described by Hammond *et al*.^2^), showed improved fertility in the *zpg*-driven constructs^17^ (summarized in Supplementary Fig. 4).

To make p17410 (GenBank accession code MH541847), we amplified both the promoter and terminator using primers carrying arms suitable for a subsequent Gibson assembly (Supplementary Table 4). The promoter, a 1,074-bp region upstream of the gene also containing the 5′ UTR, was amplified using primers zpgprCRISPR-F and zpgprCRISPR-R from the wild-type G3 mosquito strain. The terminator, a 1,037-bp region downstream also containing the 3′ UTR, was amplified using primers zpgteCRISPR-F and zpgteCRISPR-R. Using restriction enzymes, we removed the *hCas9* gene, backbone and gRNA cassette from p16510 and reassembled everything in a Gibson assembly reaction using the *zpg* promoter and terminator fragments.

### Microinjection of embryos and selection of transformed mosquitoes.

All mosquitoes were reared under standard conditions of 80% relative humidity and 28 °C. The mosquitoes were blood-fed on anesthetized mice, and freshly laid embryos were aligned and used for microinjections as described before^32^. We injected embryos with solution containing both p16510 and pK101 (each at 300 ng/μl) to generate mosquitoes (*dsxF*^*−*^) in which the splicing junction of *dsx* exon 5 had been disrupted by the insertion of the eGFP ϕC31 acceptor construct. To generate the *dsxF* CRISPR homing allele, embryos from the *dsxF*^*−*^ knock-in line were injected with solution containing p17410 and a plasmid-based source of ϕC31 integrase^2^. All the surviving G_0_ larvae were crossed to wild-type mosquitoes and G_1_ positive transformants were identified using a fluorescence microscope (Eclipse TE200) as GFP^+^ larvae for the knock-in events and RFP^+^ larvae for the RMCE events.

### Containment of gene drive mosquitoes.

All mosquitoes were housed at Imperial College London in an insectary that is compliant with Arthropod Containment Guidelines Level 2 (ref. 33). All GM work was performed under institutionally approved biosafety and GM protocols. In particular, GM mosquitoes containing constructs with the potential to show gene drive were housed in dedicated cubicles, separated by at least six doors from the external environment and requiring two levels of security card access. Moreover, because of its location in a city with a northern temperate climate, *A. gambiae* mosquitoes housed in the insectary are also ecologically contained. The physical and ecological containment of the insectary are compliant with guidelines set out in a recent commentary calling for safeguards in the study of synthetic gene drive technologies^34^.

### Molecular confirmation of gene targeting and cassette integration.

Successful integration of *dsxF*^*−*^ and *dsxF*^*CRISPRh*^ cassettes into *Agdsx* at exon 5 was confirmed by PCR using genomic DNA extracted using the Wizard Genomic DNA purification kit (Promega). Generation of the HDR-mediated *dsxF*^*−*^ allele was confirmed using primers binding the integrated cassette (GFP-F and 3xP3-R) and the neighboring genomic integration site, external to the sequence included on the homology arms (dsxin3-F and dsxex6-R). *dsxF*^*−*^ heterozygotes and homozygotes could be further distinguished by PCR using primers that bind either side of the inserted cassette (dsxex4-F and dsxex5-R), giving rise to a smaller and/or larger product corresponding to the empty wild-type locus or the predicted *dsxF*^*−*^ allele, respectively.

RCME of the *dsxF*^*CRISPRh*^ construct into the *dsx* locus was confirmed using primers binding the drive cassette (hCas9-F and RFP-R) and the neighboring genomic integration site (dsxin4-F and dsxex5-R1). Primer sequences can be found in Supplementary Table 4.

### Phenotypic characterization and microdissections.

Microdissection and phenotypic characterization were carried out using Olympus SZX7 optical microscopes. Mosquitoes were collected in Falcon tubes and anesthetized on ice 5 min before dissection. For phenotypic comparison, the legs of the mosquitoes were removed to achieve the profile orientation. Pictures were taken using a HiChrome-SMII GXCAM digital mounted camera (GT Vision). Pictures of gonads were taken using the EVOS imaging system (Thermo-Fisher).

### Phenotypic assays.

Phenotypic assays designed to examine relative fecundity in mosquitoes carrying either *dsxF*^*−*^ or *dsxF*^*CRISPRh*^ alleles were carried out essentially as described before^2^. Briefly, the offspring of intercrossed heterozygous *dsxF*^*−/+*^ individuals were screened for heterozygous or homozygous knock-in on the basis of weak or strong GFP expression, respectively. Nonfluorescent progeny were kept as controls. Groups of 50 male and 50 female mosquitoes from each of the three classes were mated to an equal number of wild-type mosquitoes for 5 d, blood-fed, and a minimum of 45 females allowed to lay individually. The entire egg and larval progeny were counted for each lay and a minimum of 20 progeny investigated to confirm zygosity of the *dsxF*^*−*^ allele in the parent. Females that failed to give progeny and had no evidence of sperm in their spermathecae were excluded from the analysis. Phenotypic assays for *dsxF*^*CRISPRh*^ individuals were performed essentially the same way with the exception that the entire larval progeny were screened for presence of DsRed, which is linked to the *dsxF*^*CRISPRh*^ allele. Statistical differences between genotypes were assessed using the Kruskal–Wallis test.

### Cage trial assays.

Two cage trials were initiated using 300 wild-type females, 150 wild-type males and 150 *dsxF*^*CRISPRh*^/+ males. The wild-type and *dsxF*^*CRISPRh*^ lines were reared in parallel and kept under the same conditions. For the starting generation only, age-matched male and female pupae were allowed to emerge in separate cages and were mixed only when all the pupae had emerged. Both *dsxF*^*CRISPRh*^ and wild-type male pupae were screened for the presence of the RFP marker. Mosquitoes were left to mate for 5 days before they were blood fed on anesthetized mice. Two days after, the mosquitoes were set to lay in a 300-ml egg bowl filled with water and lined with filter paper. The eggs produced from the cage were photographed and counted using JMicroVision V1.27. Prior to counting, eggs were dispersed using gentle water spraying in the egg bowl to homogenize the population, and 650 eggs were randomly selected to seed the next generation. Larvae emerging from the 650 eggs were counted and screened for the presence of the RFP marker to score the transgenic rate of the progeny. The number of pupae used to seed the next generation was also recorded.

### PCR of target site and deep sequencing analysis preparation.

For the deep sequence analysis, a limiting PCR reaction was performed on 40 ng of genomic material extracted *en masse* using the Wizard Genomic DNA purification kit (Promega) from a minimum of 359 mosquitoes taken at G_2_, G_3_, G_4_ and G_5_ from both cage experiments. Using the KAPA HiFi HotStart Ready Mix PCR kit (Kapa Biosystems) and primers that carried the Illumina Nextera Transposase adapters (underlined), 4050-Illumina-F (TCGTCGGCAGCGTCAGATGTGTATAAGAGACAGACTTATCGGCATCAGTTGCG) and 4050-Illumina-R (GTCTCGTGGGCTCGGAGATGTGTATAAGAGACAGGTGAATTCCGTCAGCCAGCA), we amplified a 358-bp locus containing the target site in 50-μl reactions. To maintain the proportion of the reads corresponding to particular alleles at the target site, the PCR reactions were performed under nonsaturating conditions; they were allowed to run for 20 cycles before 25 μl were removed and stored at −20 °C. The remnant 25 μl were run for another 20 cycles and used to verify the amplification on an agarose gel. Annealing time and temperature were adjusted to 68 °C for 20 s to minimize off-target amplification.

Libraries were prepared in accordance with the Illumina 16S Metagenomic Sequencing Library Preparation protocol and the Nextera XT Index Kit. AMPure XP beads were used to purify the amplicons. Dual indices and Illumina sequencing adapters were attached in a second PCR step using the Nextera XT Indexing Kit and purified with the AMPure XP beads. The resulting libraries were validated using an Agilent 2100 Bioanalyzer (DNA High Sensitivity kit, sample dilution 1:5) to determine size distribution and a Qubit 3.0 fluorometer to determine concentration of libraries. Indexed DNA libraries were normalized to 4 nM, pooled and loaded at a concentration of 9 pM onto an Illumina Flowcell v2 with 19% of ϕX control and sequenced using the Illumina MiSeq, 2 × 250 bp v2 paired end run.

### Deep sequencing analysis.

We ran CRISPResso^35^ software v1.0.8 on raw sequencing data to detect mutations at the target site using parameter -q 30, setting the minimum average read quality score (phred33) to 30. Raw sequencing data was deposited in the NCBI BioProject database (accession code PRJNA476358). Resulting allele frequency tables were processed using *ad hoc* Python and R scripts to group, filter and visualize indels and substitutions in the amplicon. To visualize the frequency of the most abundant indels around the cut site in both cages over the four generations, we calculated the mean frequency of indels occurring within the target region, including 20 bp upstream and downstream of the target site. The top ten alleles with the highest mean frequency were then selected to show the change of frequency of each allele throughout four generations. To plot and show the distribution of indels and substitutions in the whole amplicon, we filtered out alleles with less than three reads.

### Modeling.

We use discrete-generation deterministic and stochastic models with random mating and males and females treated separately as in Hammond *et al*.^9^, and incorporate different homing rates in males and females and a modified treatment of embryonic cleavage and repair from paternally and maternally derived nuclease, as observed (see “Population genetics model” below^9,36^). We include wild-type (W), driver (D), and nonfunctional nuclease-resistant (R) alleles. Cleavage followed by homing and repair occurs in the germline in heterozygous W/D females and males; otherwise inheritance is Mendelian. Gametes (W, D or R) from W/D females and W/D, D/R and D/D males carry nuclease that is transmitted to the zygote and acts in the embryo in somatic cells to reduce fitness if wild-type alleles are present, so that W/W, W/R and W/D females have fitness w10, w01, w11 or 1, depending on whether nuclease was derived from a transgenic mother, father, both or neither. All males are assumed to have fitness 1, and we assume no effects of parentally deposited nuclease in germline cells. In the stochastic version of the model, probabilities of mating, egg production, hatching and emergence from pupae are estimated from experiments (Supplementary Table 5) and random numbers for these events are taken from the appropriate multinomial distributions. To model the cage experiments, 300 females and 150 male wild-type adults along with 150 male drive heterozygotes (from transgenic fathers) are initially present. Females may fail to mate or may mate once randomly with a male of a given genotype according to its frequency in the male population. The number of eggs produced from each mated female is randomly chosen by sampling with replacement from experimental values in Supplementary Table 6. To start the next generation, 650 eggs are randomly selected, and these hatch with a probability that also depends upon on the genotype of the mother. The probability of subsequent survival to adulthood is assumed to be equal across genotypes.

### Population genetics model.

To model the results of the cage experiments, we use discrete-generation recursion equations for the genotype frequencies, treating males and females separately. *F*_*ij*_(*t*) and *M*_*ij*_(*t*) denote the frequency of females (or males) of genotype *i*/*j* in the total female (or male) population. We consider three alleles, W (wild-type), D (driver) and R (nonfunctional resistant), and therefore six genotypes.

*Homing*. Adults of genotype W/D produce gametes at meiosis in the ratio W:D:R as follows:

Here *d*_f_ and *d*_m_ are the rates of transmission of the driver allele in the two sexes and *u*_f_ and *u*_m_ are the fractions of nondrive gametes that are nonfunctional resistant (R alleles) from meiotic end-joining. In all other genotypes, inheritance is Mendelian.

*Fitness*. Let *w*_*ij*_ ≤ 1 represent the fitness of genotype *i*/*j* relative to *w*_WW_ = 1 for the wild-type homozygote. We assume no fitness effects in males. Fitness effects in females are manifested as differences in the relative ability of genotypes to participate in mating and reproduction. We assume the target gene is needed for female fertility, and thus D/D, D/R and R/R females are sterile; there is no reduction in fitness in females with only one copy of the target gene (W/D, W/R).

*Parental effects*. We consider that further cleavage of the W allele and repair can occur in the embryo if nuclease is present, due to one or both contributing gametes derived from a parent with one or two driver alleles. The presence of parental nuclease is assumed to affect somatic cells and therefore female fitness but has no effect in germline cells that would alter gene transmission. Previously, embryonic EJ effects (maternal only) were modeled as acting immediately in the zygote. Here, we consider that experimental measurements of female individuals of different genotypes and origins show a range of fitnesses, suggesting that individuals may be mosaics with intermediate phenotypes. We therefore model genotypes W/X (X = W, D, R) with parental nuclease as individuals with an intermediate reduced fitness ,  or  depending on whether nuclease was derived from a transgenic mother, father or both. We assume that parental effects are the same whether the parent(s) had one or two drive alleles. For simplicity, a baseline reduced fitness of *w*_10_, *w*_01_, *w*_11_ is assigned to all genotypes W/X (X = W, D, R) with maternal, paternal and maternal/paternal effects, with fitness estimated as the product of mean egg production values and hatching rates relative to wild type in Supplementary Table 5 in the deterministic model. In the stochastic version of the model, egg production from female individuals with different parentage is sampled with replacement from experimental values.

*Recursion equations*. We first consider the gamete contributions from each genotype, including parental effects on fitness. In addition to W and R gametes that are derived from parents that have no drive allele and therefore have no deposited nuclease, gametes from W/D females and W/D, D/R and D/D males carry nuclease that is transmitted to the zygote, and these are denoted W^*^, D^*^ and R^*^. The proportion *e*_*i*_ of type *i* alleles in eggs produced by females participating in reproduction are given in terms of male and female genotype frequencies below. Frequencies of mosaic individuals with parental effects (i.e., reduced fitness) due to nuclease from mothers, fathers or both are denoted by superscripts 10, 01 or 11.

The proportions *s*_*i*_ of type *i* alleles in sperm are

Above,  and  are the average female and male fitness:

To model cage experiments, we start with an equal number of males and females, with an initial frequency of wild-type females in the female population of *F*_WW_ = 1, wild-type males in the male population of *M*_WW_ = 1/2, and  heterozygote drive males that inherited the drive from their fathers. Assuming a 1:1 ratio of males and females in progeny, after the starting generation, genotype frequencies of type *i*/*j* in the next generation (*t* + 1) are the same in males and females, *F*_*ij*_(*t* + 1) = *M*_*ij*_(*t* + 1). Both are given by *G*_*ij*_(*t* + 1) in the following set of equations in terms of the gamete proportions in the previous generation, assuming random mating:

The frequency of transgenic individuals can be compared with experiment: the fraction of RFP^+^ individuals is given by

All calculations are carried out using Wolfram Mathematica (Wolfram Research Inc.)

### *In vitro* cleavage assay against wild-type and SNP variant target site.

We performed an *in vitro* cleavage assay to test the ability of the gRNA used in this study to cleave the target site that incorporates the SNP found in wild populations in Africa (Supplementary Fig. 9). Using Golden Gate cloning and primers modified to carry suitable overhangs, we introduced the two target sequences separately into a 2-kb plasmid. As a control, we also prepared a plasmid that carries a modified version of the *dsx* target site without the SNP that lacks the PAM sequence, necessary for Cas9 cleavage. All three vectors were linearized and verified on a gel before the cleavage assay. For the cleavage assay we used a ready-to-use sgRNA provided by Synthego (USA) and *S. pyogenes* Cas9 nuclease in the form of enzyme (NEB). To form ribonucleoprotein particles (RNPs), we mixed a 1:1 molar ration of the sgRNA and the Cas9 protein into a 40-μl reaction to a final concentration of 400 nM and left it to incubate at room temperature for 10 min. The linearized substrate was added to the reactions in a final concentration of 40 nM in a final volume of 50 μl and incubated at 37 °C for 30 min. Proteinase K was added to stop the reaction and 20 μl were verified on a gel. The primers used to create the three target sequences are outlined in Supplementary Table 4.

### Ethics statement.

All animal work was conducted according to UK Home Office Regulations and approved under Home Office License PPL 70/8914.

### Life Sciences Reporting Summary.

Further information on research design is available in the Nature Research Reporting Summary linked to this article.

### Data availability.

Raw sequencing data were deposited in the NCBI BioProject database under accession code PRJNA476358.

## Additional information

**Publisher's note:** Springer Nature remains neutral with regard to jurisdictional claims in published maps and institutional affiliations.

## Supplementary Information

### Integrated supplementary information

Supplementary Figure 1 Molecular confirmation of the correct integration of the HDR-mediated event to generate *dsxF* PCRs were performed to verify the location of the *dsx* ϕC31 knock-in integration. Primers (blue arrows) were designed to bind internal of the ϕC31 construct and outside of the regions used for homology directed repair (HDR) (dotted grey lines) which were included in the Donor plasmid K101. Amplicons of the expected sizes should only be produced in the event of a correct HDR integration. The gel shows PCRs performed on the 5’ (left) and 3’ (right) of 3 individuals for the *dsx* ϕC31 knock-in line (*dsxF*^*−*^) and wild type (wt) as a negative control.

Supplementary Figure 2 Morphology of the *dsxF*^*−/−*^ internal reproductive organs (**a**) Testis-like gonad from 3-days old female *dsxF*^*−/−*^ individual. There was no layer division between the cells and there was no evidence of sperm. (**b**) Dissections performed on *dsxF*^*−/−*^ genetic females revealed the presence of organs resembling accessory glands, a typical male internal reproductive organ. (**c**) somatic mosaicism of penetrance of *dsxF*^*−/−*^ phenotype in *dsxF*^*CRISPRh*^/+ females due to paternal deposition of nuclease, that can result in partial formation of clasper sets.

Supplementary Figure 3 Development of *dsxF*^*CRISPRh*^ drive construct and its predicted homing process and molecular confirmation of the locus (**a**) The drive construct (*CRISPR*^*h*^ cassette) contained the transcription unit of a human codon-optimised *Cas9* controlled by the germline-restrictive *zpg* promoter, the RFP gene under the control of the neuronal *3xP3* promoter and the gRNA under the control of the constitutive *U6* promoter, all enclosed within two *attB* sequences. The cassette was inserted at the target locus using recombinase-mediated cassette exchange (RMCE) by injecting embryos with a plasmid containing the cassette and a plasmid containing a ϕC31 recombination transcription unit. During meiosis the Cas9/gRNA complex cleaves the wild-type allele at the target locus (DSB) and the construct is copied across to the wild-type allele via HDR (homing) disrupting exon 5 in the process. (**b**) Representative example of molecular confirmation of successful RMCE events. Primers (blue arrows) that bind components of the *CRISPR*^*h*^ cassette were combined with primers that bind the genomic region surrounding the construct. PCRs were performed on both sides of the *CRISPR*^*h*^ cassette (5’ and 3’) on many individuals as well as wild-type controls (wt).

Supplementary Figure 4 Gene drives designed to express Cas9 under regulation of the promoter and terminator regions of *zpg* show high rates of biased transmission and substantially improved fertility compared with the *vasa*2 promoter at a previously validated female fertility locus (*AGAP007280*) Phenotypic assays were performed to measure fertility and transmission rates for each gene drive based upon the *vasa* and *zpg* promoters. The data for the *vasa-CRISPR*^*h*^ is previously reported in Hammond et al. (2016). The *zpg-CRISPR*^*h*^ construct targeting AGAP007280 recognised exactly the same target site and was inserted in identical fashion to the *vasa-CRISPR*^*h*^, through recombinase-mediated cassette exchange^9^. The larval output was determined for individual drive heterozygotes crossed to wild type (left), and their progeny scored for the presence of DsRed linked to the construct (right). The average progeny count and transmission rate is also shown (± s.e.m.).

Supplementary Figure 5 Maternal or paternal inheritance of the *dsxF*^*CRISPRh*^ driving allele affect fecundity and transmission bias in heterozygotes Male and female *dsxF*^*CRISPRh*^ heterozygotes (*dsxF*^*CRISPRh*^/+) that had inherited a maternal or paternal copy of the driving allele were crossed to wild type and assessed for inheritance bias of the construct (**a**) and reproductive phenotype (**b**). (**a**) Progeny from single crosses (n≥15) were screened for the fraction that inherited DsRed marker gene linked to the *dsxF*^*CRISPRh*^ driving allele (e.g. G_1_♂→G_2_♀ represents a heterozygous female that received the drive allele from her father). Levels of homing were similarly high in males and females whether the allele had been inherited maternally or paternally. The dotted line indicates the expected Mendelian inheritance. Mean transmission rate (± s.e.m.) is shown. (**b**) Counts of hatched larvae for the individual crosses revealed a fertility cost in female *dsxF*^*CRISPRh*^ heterozygotes that was stronger when the allele was inherited paternally. Mean progeny count (± s.e.m.) is shown. (***, p<0.001;****, p<0.0001; Kruskal-Wallis test).

Supplementary Figure 6 Probability of stochastic loss of the drive as a function of initial number of male drive heterozygotes To calculate the probability of stochastic loss of the drive in the cage experiment setup, for each initial number (h0) of male drive heterozygous individuals, out of 1000 simulations of the stochastic cage model (described in Supp Info), we recorded the number of times the drive was not present at 40 generations (and consequently population elimination did not occur). Each data point represents 1000 individual simulations of the stochastic cage model.

Supplementary Figure 7 Frequency plots of variants and indels in target sequence Pooled amplicon sequencing of the target site from 4 generations of the cage experiment (generations 2, 3, 4 and 5) revealed a range of very low frequency indels at the target site (**a**), none of which showed any sign of positive selection. Insertion, deletion and substitution frequencies per nucleotide position were calculated, as a fraction of all non-drive alleles, from the deep sequencing analysis for both cages. Distribution of insertions and deletions (**b**) in the amplicon is shown for each cage. Contribution of insertions and deletions arising from different generations is displayed with the frequency in each generation represented by a different colour. Significant change (p<0.01) in the overall indel frequency was observed in the region around the cut-site (dotted area ± 20 bp) for both cages. No significant changes were observed in the substitution frequency (**c**) around the cut-site (shaded area ± 20 bp) when compared with the rest of the amplicon, confirming that the gene drive did not generate any substitution activity at the target locus and that the laboratory colony is devoid of any standing variation in the form of SNPs within the entire amplicon.

Supplementary Figure 8 Sequence comparison of the *dsx* female-specific exon 5 across members of the *Anopheles* genus and SNP data obtained from *A. gambiae* mosquitoes in Africa. (**a**) Sequence comparison of the *dsx* intron 4-exon 5 boundary and the *dsx* female-specific exon 5 within the 16 anopheline species^16^. The sequence of the intron 4-exon 5 boundary is completely conserved within the six species that form the *Anopheles gambiae* species complex (noted in **bold**). The gRNA used to target the gene is underlined and the PAM is highlighted in blue. Changes in the DNA sequence are shaded grey and codon silent and missense substitutions are noted in blue and red respectively. (**b**) SNP frequencies obtained from 765 *Anopheles gambiae* mosquitoes captured across Africa^17^. Across the *dsx* female-specific Exon 5 there are only 2 SNP variants (noted in yellow) with frequencies of 2.9% (the SNP in the gRNA-complementary sequence) and 0.07%.

Supplementary Figure 9 *In vitro* cleavage assay testing the efficiency of the gRNA in the *dsxF*^*CRISPRh*^ gene drive to cleave the *dsx* exon 5 target site with the SNP found in wild populations in Africa An in vitro cleavage assay using an RNP complex of Cas9 enzyme and the gRNA used in this study was performed against linearised plasmids containing either wild-type (WT) target site in *dsx* exon 5 or the same site containing the single SNP found in wild caught populations (SNP). Products of the in vitro cleavage assay were purified and analysed on a gel. Both the WT and SNP-containing target sites are susceptible to the cleavage activity of the RNP complex as shown by the diminished high molecular band and the presence of the two cleavage products of the expected size. A *dsx* exon 5 target site containing the WT sequence complementary to the gRNA but without the PAM sequence was used as a control (‘no PAM’).

Supplementary Figure 10 Modeling the effect of unforeseen additional fitness reduction encountered by heterozygous gene drive females Time dynamics of drive allele frequency as predicted by the deterministic model, with different coloured lines representing additional percentage reductions from zero to 100% in the baseline fertility of females, mimicking an ecologically more realistic scenario in which there were more severe fitness effects associated with the gene drive than in the laboratory. The reduction in fitness is assumed to affect the overall reproductive success (i.e mating success, longevity, fertility etc.). The baseline fertility values relative to the wild type are those reported in Supplementary Table S4 (0.65 for females with transgenic mothers, and 0.217 for females with transgenic fathers), that describes these and all other parameters estimated from experiment. Fitness reductions of up to 40% are predicted to crash the population. The spread of the drive is computed using the deterministic model in Figure 5.

### Supplementary information

Supplementary Text and Figures Supplementary Figures 1–10 (PDF 1567 kb)

Life Sciences Reporting Summary (PDF 129 kb)

Supplementary Tables Supplementary Tables 1–6 (PDF 1046 kb)

## Data Availability

BioProject
PRJNA476358 NCBI Reference Sequence
MH541846
MH541847
