# Supplementary figures and images for: A CRISPR–Cas9 gene drive targeting doublesex causes complete population suppression in caged Anopheles gambiae mosquitoes
Source: Nat Biotechnol. 2018 Sep 24;36(11):1062–6. doi: 10.1038/nbt.4245 (PMC6871539; doi:10.1038/nbt.4245)

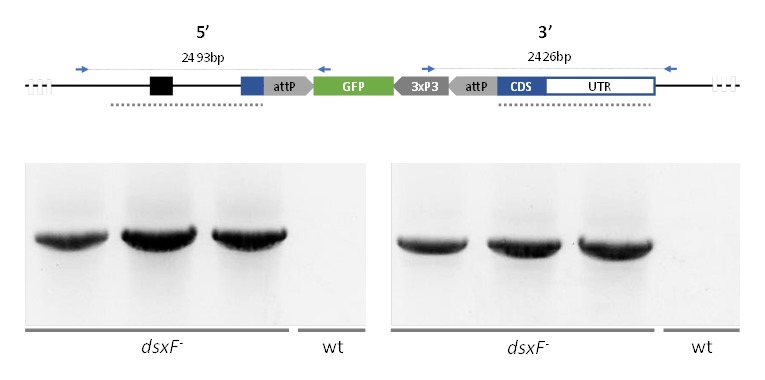

Supplement: Molecular confirmation of the correct integration of the HDR-mediated event to generate dsxF — PCRs were performed to verify the location of the dsx ϕC31 knock-in integration. Primers (blue arrows) were designed to bind internal of the ϕC31 construct and outside of the regions used for homology directed repair (HDR) (dotted grey lines) which were included in the Donor plasmid K101. Amplicons of the expected sizes should only be produced in the event of a correct HDR integration. The gel shows PCRs performed on the 5’ (left) and 3’ (right) of 3 individuals for the dsx ϕC31 knock-in line (dsxF−) and wild type (wt) as a negative control. [file 41587_2018_Article_BFnbt4245_Fig6_ESM.jpg]

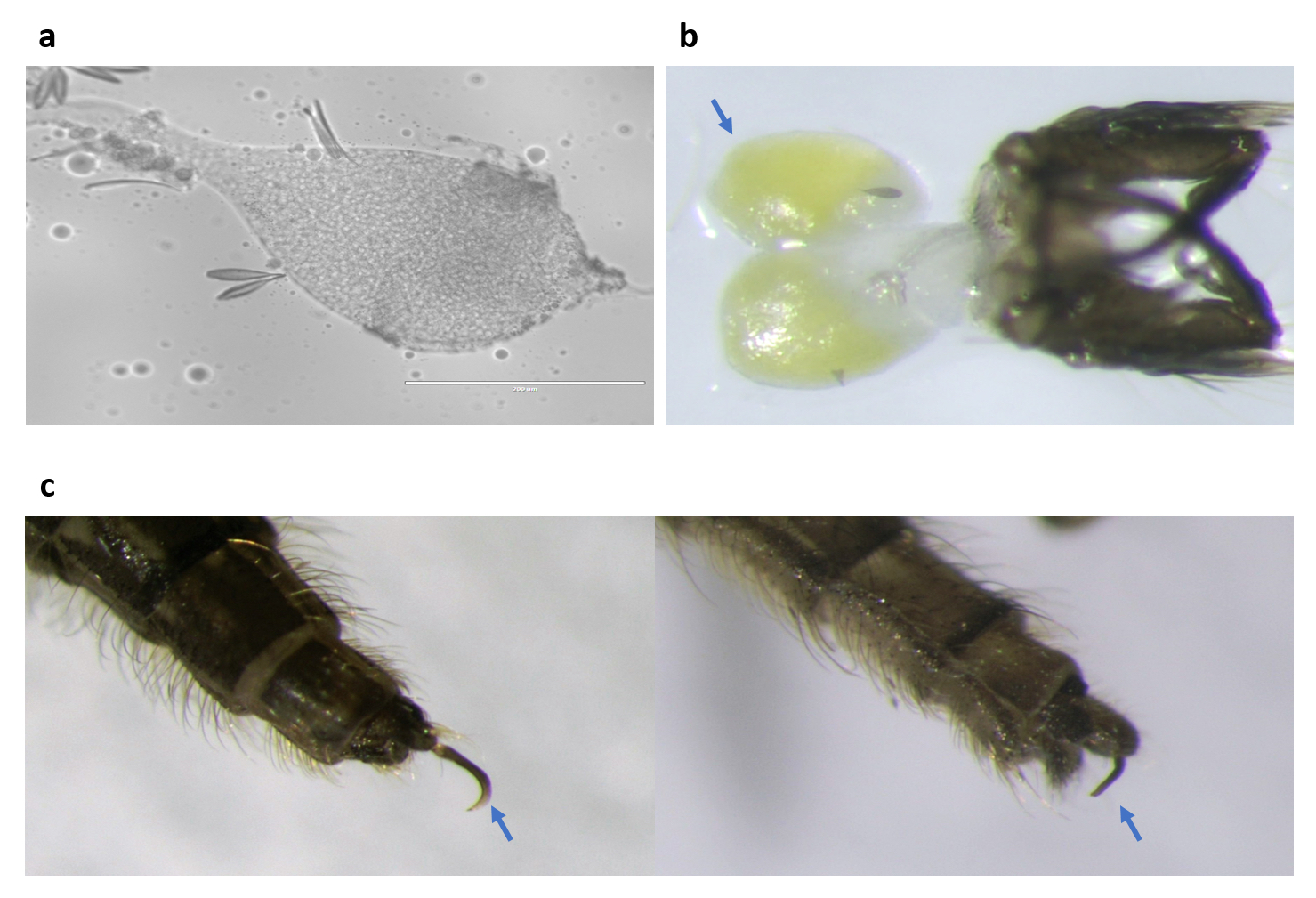

Supplement: Morphology of the dsxF−/− internal reproductive organs — (a) Testis-like gonad from 3-days old female dsxF−/− individual. There was no layer division between the cells and there was no evidence of sperm. (b) Dissections performed on dsxF−/− genetic females revealed the presence of organs resembling accessory glands, a typical male internal reproductive organ. (c) somatic mosaicism of penetrance of dsxF−/− phenotype in dsxFCRISPRh/+ females due to paternal deposition of nuclease, that can result in partial formation of clasper sets. [file 41587_2018_Article_BFnbt4245_Fig7_ESM.jpg]

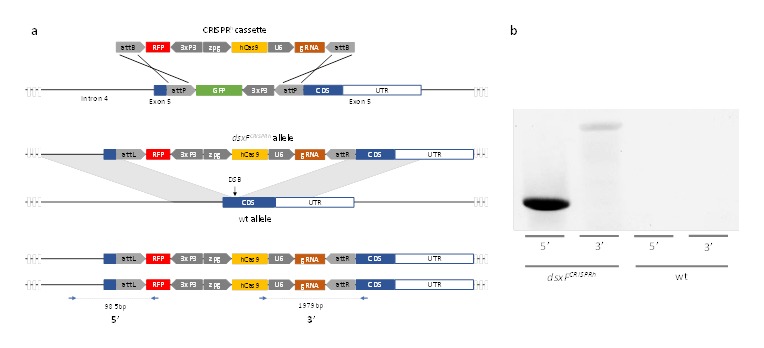

Supplement: Development of dsxFCRISPRh drive construct and its predicted homing process and molecular confirmation of the locus — (a) The drive construct (CRISPRh cassette) contained the transcription unit of a human codon-optimised Cas9 controlled by the germline-restrictive zpg promoter, the RFP gene under the control of the neuronal 3xP3 promoter and the gRNA under the control of the constitutive U6 promoter, all enclosed within two attB sequences. The cassette was inserted at the target locus using recombinase-mediated cassette exchange (RMCE) by injecting embryos with a plasmid containing the cassette and a plasmid containing a ϕC31 recombination transcription unit. During meiosis the Cas9/gRNA complex cleaves the wild-type allele at the target locus (DSB) and the construct is copied across to the wild-type allele via HDR (homing) disrupting exon 5 in the process. (b) Representative example of molecular confirmation of successful RMCE events. Primers (blue arrows) that bind components of the CRISPRh cassette were combined with primers that bind the genomic region surrounding the construct. PCRs were performed on both sides of the CRISPRh cassette (5’ and 3’) on many individuals as well as wild-type controls (wt). [file 41587_2018_Article_BFnbt4245_Fig8_ESM.jpg]

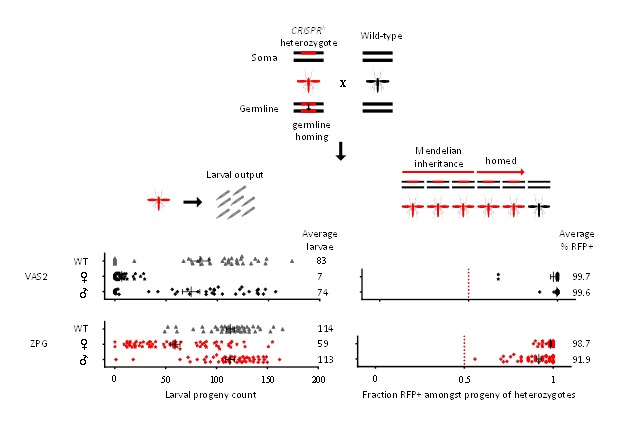

Supplement: Gene drives designed to express Cas9 under regulation of the promoter and terminator regions of zpg show high rates of biased transmission and substantially improved fertility compared with the vasa2 promoter at a previously validated female fertility locus (AGAP007280) — Phenotypic assays were performed to measure fertility and transmission rates for each gene drive based upon the vasa and zpg promoters. The data for the vasa-CRISPRh is previously reported in Hammond et al. (2016). The zpg-CRISPRh construct targeting AGAP007280 recognised exactly the same target site and was inserted in identical fashion to the vasa-CRISPRh, through recombinase-mediated cassette exchange9. The larval output was determined for individual drive heterozygotes crossed to wild type (left), and their progeny scored for the presence of DsRed linked to the construct (right). The average progeny count and transmission rate is also shown (± s.e.m.). [file 41587_2018_Article_BFnbt4245_Fig9_ESM.jpg]

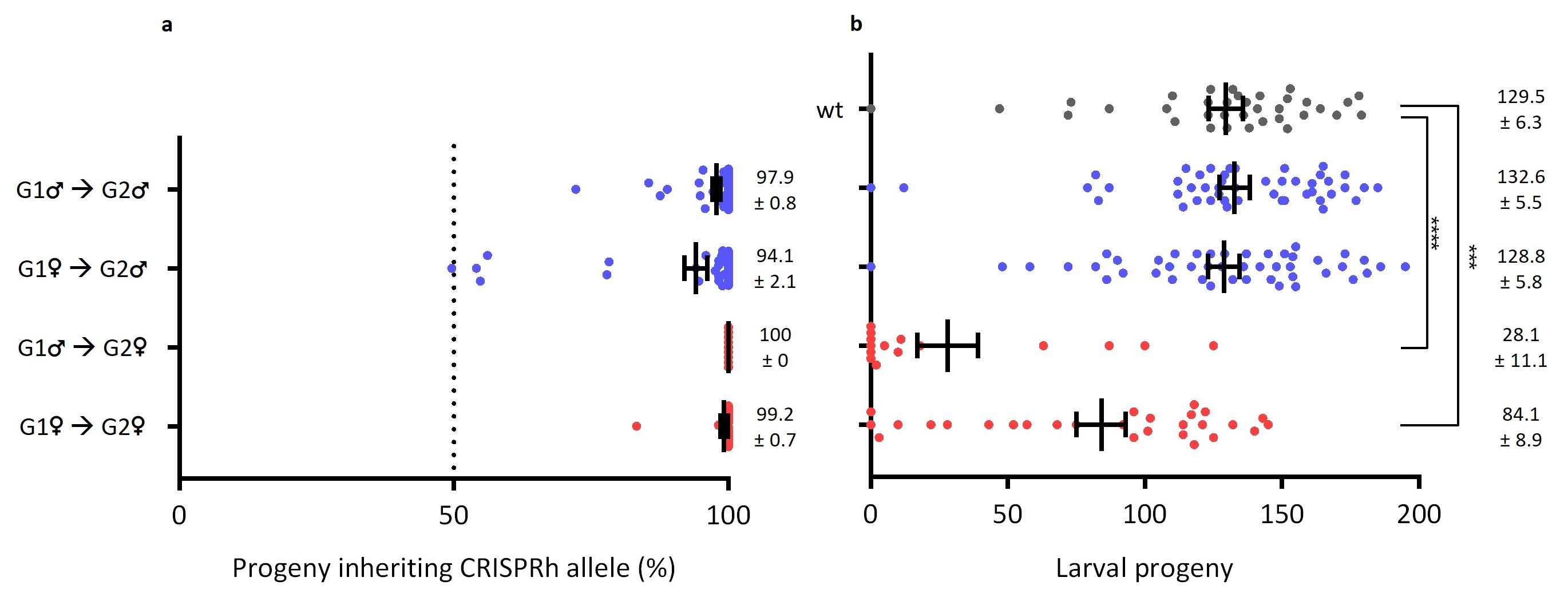

Supplement: Maternal or paternal inheritance of the dsxFCRISPRh driving allele affect fecundity and transmission bias in heterozygotes — Male and female dsxFCRISPRh heterozygotes (dsxFCRISPRh/+) that had inherited a maternal or paternal copy of the driving allele were crossed to wild type and assessed for inheritance bias of the construct (a) and reproductive phenotype (b). (a) Progeny from single crosses (n≥15) were screened for the fraction that inherited DsRed marker gene linked to the dsxFCRISPRh driving allele (e.g. G1♂→G2♀ represents a heterozygous female that received the drive allele from her father). Levels of homing were similarly high in males and females whether the allele had been inherited maternally or paternally. The dotted line indicates the expected Mendelian inheritance. Mean transmission rate (± s.e.m.) is shown. (b) Counts of hatched larvae for the individual crosses revealed a fertility cost in female dsxFCRISPRh heterozygotes that was stronger when the allele was inherited paternally. Mean progeny count (± s.e.m.) is shown. (***, p<0.001;****, p<0.0001; Kruskal-Wallis test). [file 41587_2018_Article_BFnbt4245_Fig10_ESM.jpg]

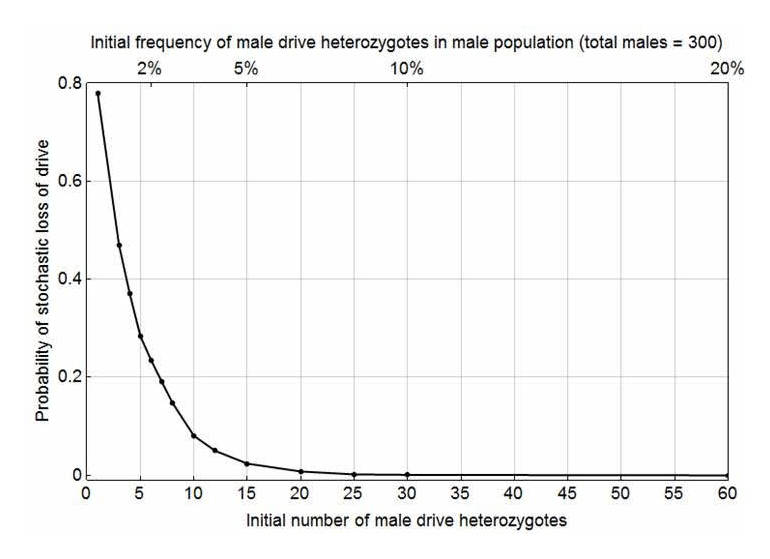

Supplement: Probability of stochastic loss of the drive as a function of initial number of male drive heterozygotes — To calculate the probability of stochastic loss of the drive in the cage experiment setup, for each initial number (h0) of male drive heterozygous individuals, out of 1000 simulations of the stochastic cage model (described in Supp Info), we recorded the number of times the drive was not present at 40 generations (and consequently population elimination did not occur). Each data point represents 1000 individual simulations of the stochastic cage model. [file 41587_2018_Article_BFnbt4245_Fig11_ESM.jpg]

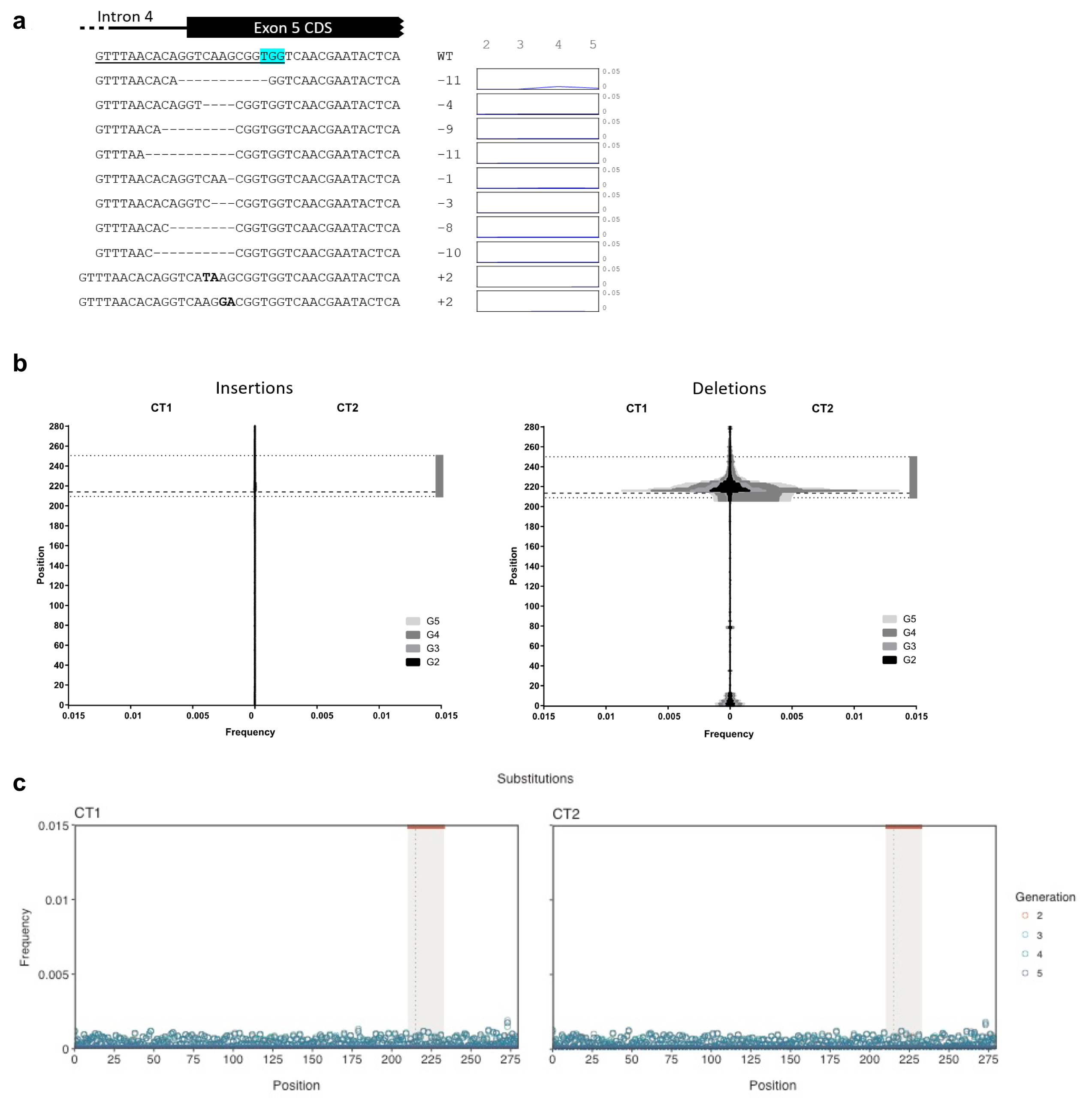

Supplement: Frequency plots of variants and indels in target sequence — Pooled amplicon sequencing of the target site from 4 generations of the cage experiment (generations 2, 3, 4 and 5) revealed a range of very low frequency indels at the target site (a), none of which showed any sign of positive selection. Insertion, deletion and substitution frequencies per nucleotide position were calculated, as a fraction of all non-drive alleles, from the deep sequencing analysis for both cages. Distribution of insertions and deletions (b) in the amplicon is shown for each cage. Contribution of insertions and deletions arising from different generations is displayed with the frequency in each generation represented by a different colour. Significant change (p<0.01) in the overall indel frequency was observed in the region around the cut-site (dotted area ± 20 bp) for both cages. No significant changes were observed in the substitution frequency (c) around the cut-site (shaded area ± 20 bp) when compared with the rest of the amplicon, confirming that the gene drive did not generate any substitution activity at the target locus and that the laboratory colony is devoid of any standing variation in the form of SNPs within the entire amplicon. [file 41587_2018_Article_BFnbt4245_Fig12_ESM.jpg]

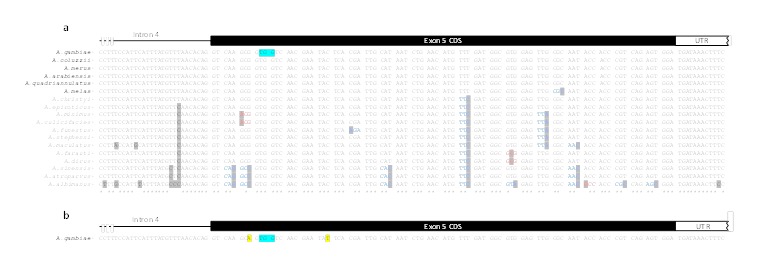

Supplement: Sequence comparison of the dsx female-specific exon 5 across members of the Anopheles genus and SNP data obtained from A. gambiae mosquitoes in Africa. — (a) Sequence comparison of the dsx intron 4-exon 5 boundary and the dsx female-specific exon 5 within the 16 anopheline species16. The sequence of the intron 4-exon 5 boundary is completely conserved within the six species that form the Anopheles gambiae species complex (noted in bold). The gRNA used to target the gene is underlined and the PAM is highlighted in blue. Changes in the DNA sequence are shaded grey and codon silent and missense substitutions are noted in blue and red respectively. (b) SNP frequencies obtained from 765 Anopheles gambiae mosquitoes captured across Africa17. Across the dsx female-specific Exon 5 there are only 2 SNP variants (noted in yellow) with frequencies of 2.9% (the SNP in the gRNA-complementary sequence) and 0.07%. [file 41587_2018_Article_BFnbt4245_Fig13_ESM.jpg]

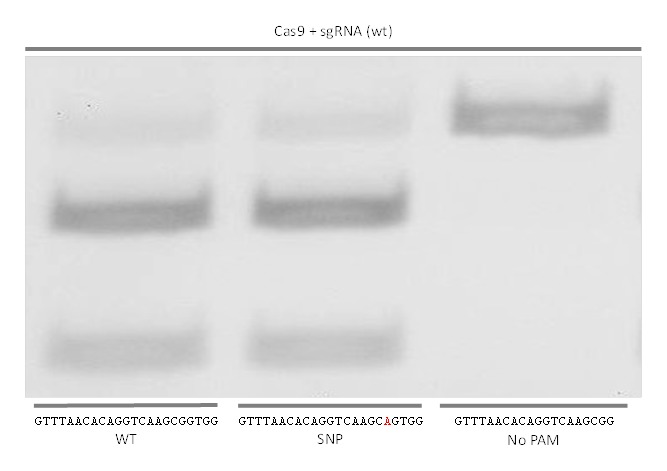

Supplement: In vitro cleavage assay testing the efficiency of the gRNA in the dsxFCRISPRh gene drive to cleave the dsx exon 5 target site with the SNP found in wild populations in Africa — An in vitro cleavage assay using an RNP complex of Cas9 enzyme and the gRNA used in this study was performed against linearised plasmids containing either wild-type (WT) target site in dsx exon 5 or the same site containing the single SNP found in wild caught populations (SNP). Products of the in vitro cleavage assay were purified and analysed on a gel. Both the WT and SNP-containing target sites are susceptible to the cleavage activity of the RNP complex as shown by the diminished high molecular band and the presence of the two cleavage products of the expected size. A dsx exon 5 target site containing the WT sequence complementary to the gRNA but without the PAM sequence was used as a control (‘no PAM’). [file 41587_2018_Article_BFnbt4245_Fig14_ESM.jpg]

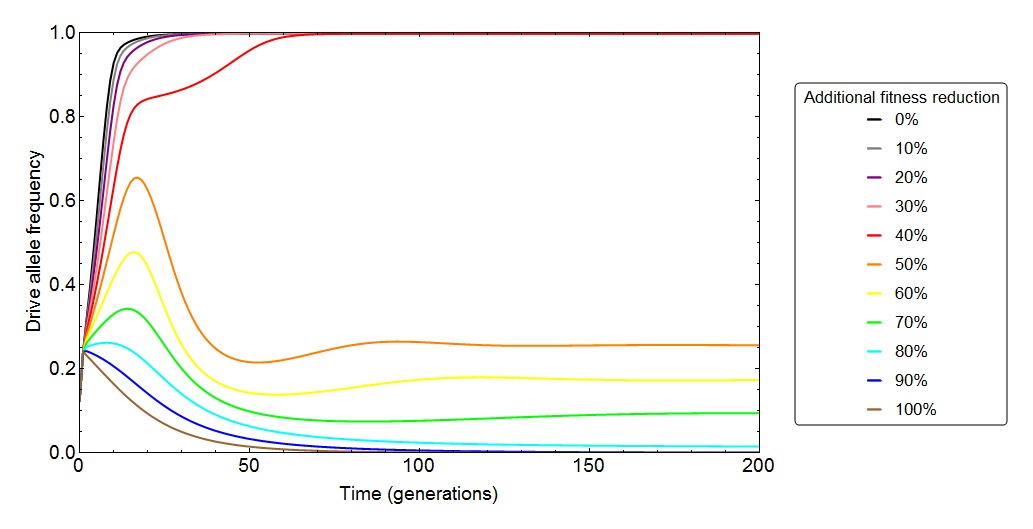

Supplement: Modeling the effect of unforeseen additional fitness reduction encountered by heterozygous gene drive females — Time dynamics of drive allele frequency as predicted by the deterministic model, with different coloured lines representing additional percentage reductions from zero to 100% in the baseline fertility of females, mimicking an ecologically more realistic scenario in which there were more severe fitness effects associated with the gene drive than in the laboratory. The reduction in fitness is assumed to affect the overall reproductive success (i.e mating success, longevity, fertility etc.). The baseline fertility values relative to the wild type are those reported in Supplementary Table S4 (0.65 for females with transgenic mothers, and 0.217 for females with transgenic fathers), that describes these and all other parameters estimated from experiment. Fitness reductions of up to 40% are predicted to crash the population. The spread of the drive is computed using the deterministic model in Figure 5. [file 41587_2018_Article_BFnbt4245_Fig15_ESM.jpg]
