# Supplementary material for: A CRISPR–Cas9 gene drive targeting doublesex causes complete population suppression in caged Anopheles gambiae mosquitoes
Source: Nat Biotechnol. 2018 Sep 24;36(11):1062–6. doi: 10.1038/nbt.4245 (PMC6871539; doi:10.1038/nbt.4245)
Supplement: Supplementary Text and Figures — Supplementary Figures 1–10 (PDF 1567 kb) [file 41587_2018_BFnbt4245_MOESM36_ESM.pdf]

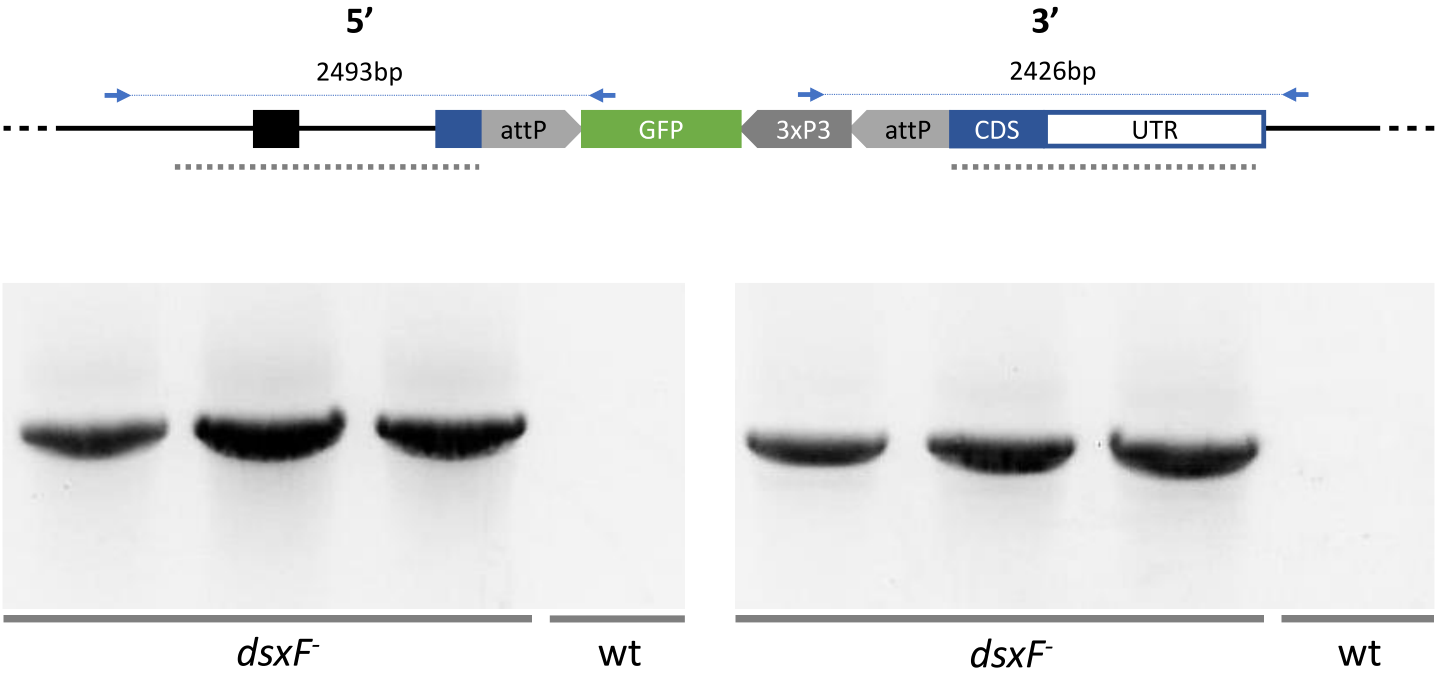

Supplementary Figure 1

Molecular confirmation of the correct integration of the HDR-mediated event to generate *dsxF*

PCRs were performed to verify the location of the *dsx* ϕC31 knock-in integration. Primers (blue arrows) were designed to bind internal of the ϕC31 construct and outside of the regions used for homology directed repair (HDR) (dotted grey lines) which were included in the Donor plasmid K101. Amplicons of the expected sizes should only be produced in the event of a correct HDR integration. The gel shows PCRs performed on the 5’ (left) and 3’ (right) of 3 individuals for the *dsx* ϕC31 knock-in line (*dsxF*$^-$) and wild type (wt) as a negative control.

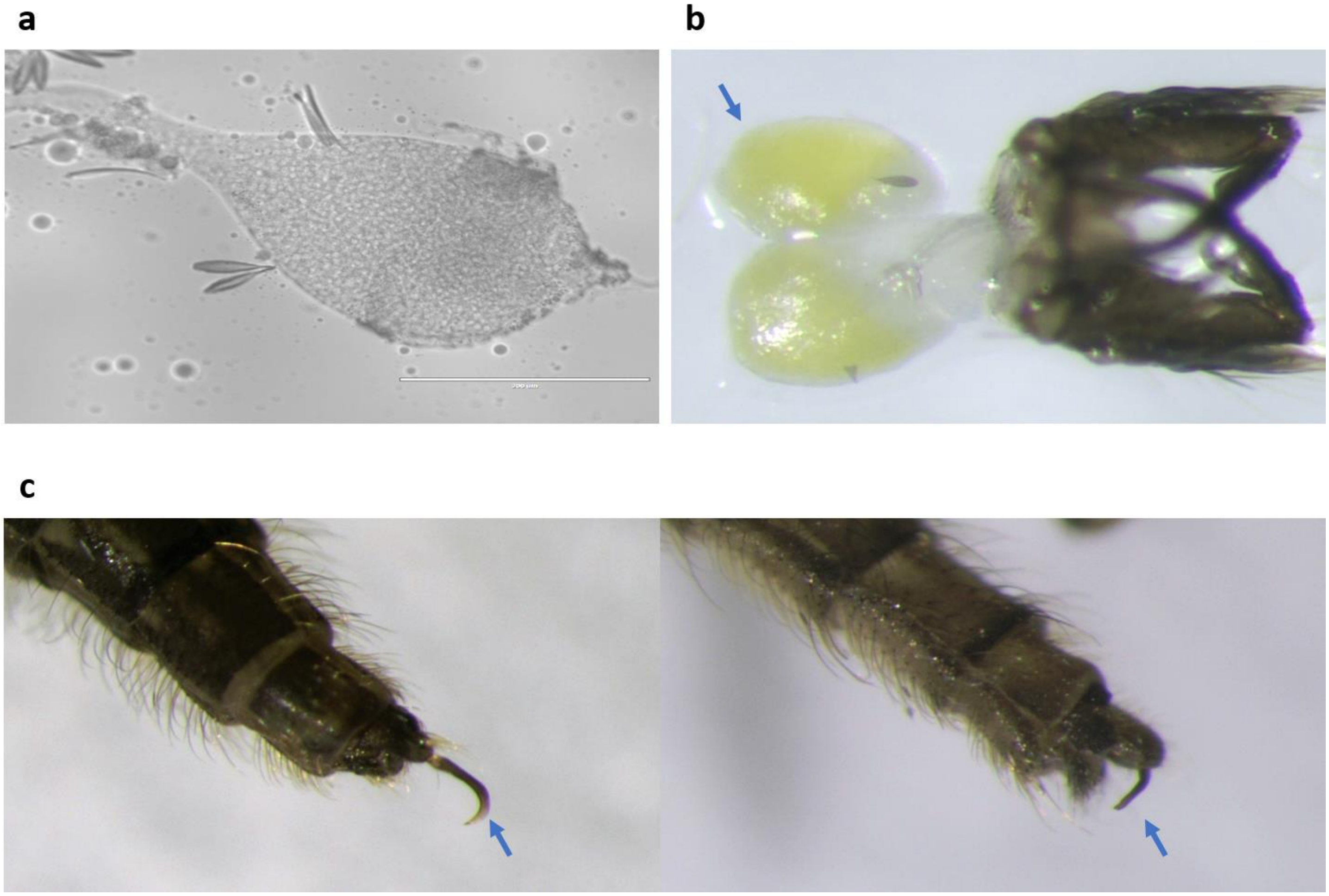

Supplementary Figure 2

Morphology of the *dsxF*$^{-/-}$ internal reproductive organs

(**a**) Testis-like gonad from 3-days old female *dsxF*$^{-/-}$ individual. There was no layer division between the cells and there was no evidence of sperm. (**b**) Dissections performed on *dsxF*$^{-/-}$ genetic females revealed the presence of organs resembling accessory glands, a typical male internal reproductive organ. (**c**) somatic mosaicism of penetrance of *dsxF*$^{-/-}$ phenotype in *dsxF*^{CRISPRh}/+ females due to paternal deposition of nuclease, that can result in partial formation of clasper sets.

Nature Biotechnology: doi:10.1038/nbt.4245

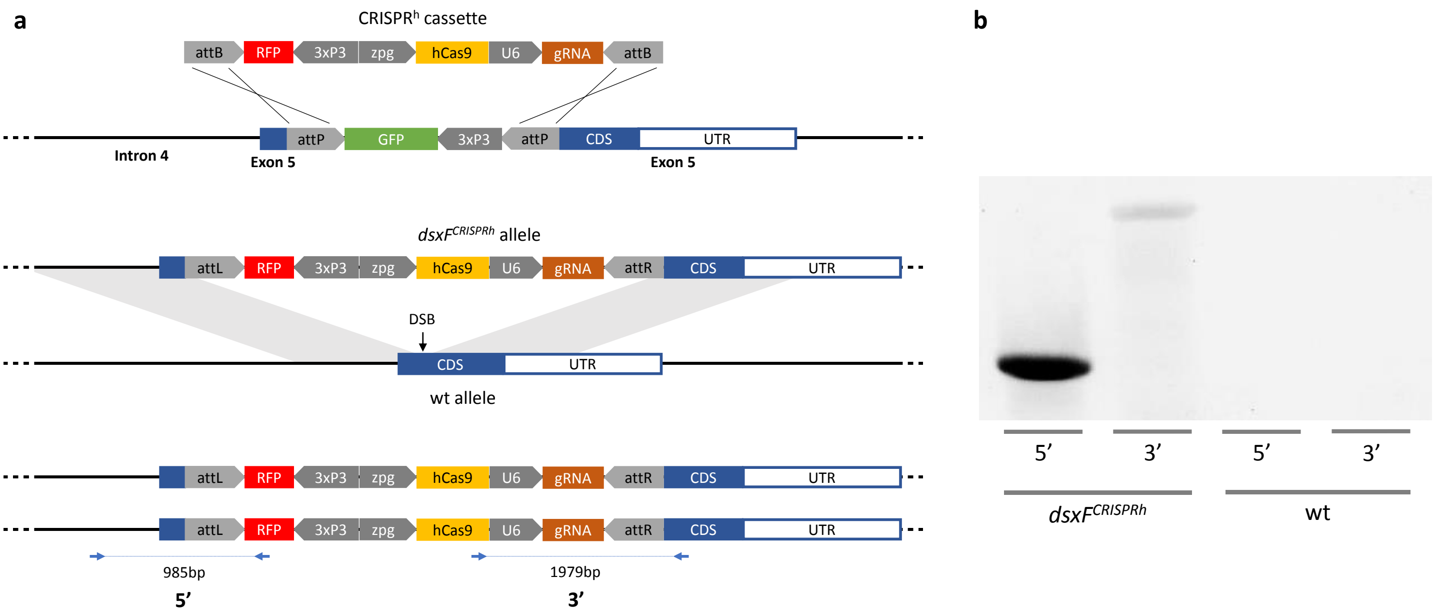

Supplementary Figure 3

Development of *dsxF*^{CRISPRh} drive construct and its predicted homing process and molecular confirmation of the locus

(**a**) The drive construct (*CRISPR*^{h} cassette) contained the transcription unit of a human codon-optimised *Cas9* controlled by the germline-restrictive *zpg* promoter, the RFP gene under the control of the neuronal *3xP3* promoter and the gRNA under the control of the constitutive *U6* promoter, all enclosed within two *attB* sequences. The cassette was inserted at the target locus using recombinase-mediated cassette exchange (RMCE) by injecting embryos with a plasmid containing the cassette and a plasmid containing a ϕC31 recombination transcription unit. During meiosis the Cas9/gRNA complex cleaves the wild-type allele at the target locus (DSB) and the construct is copied across to the wild-type allele via HDR (homing) disrupting exon 5 in the process. (**b**) Representative example of molecular confirmation of successful RMCE events. Primers (blue arrows) that bind components of the *CRISPR*^{h} cassette were combined with primers that bind the genomic region surrounding the construct. PCRs were performed on both sides of the *CRISPR*^{h} cassette (5' and 3') on many individuals as well as wild-type controls (wt).

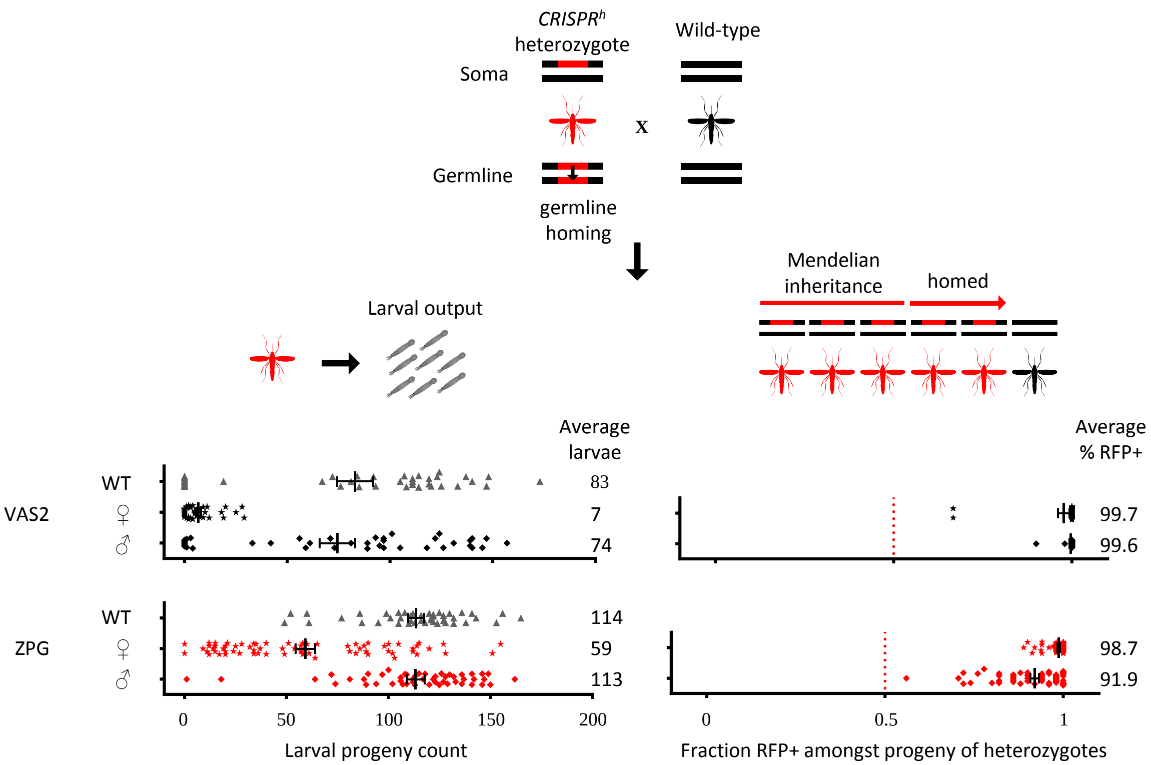

Supplementary Figure 4

Gene drives designed to express Cas9 under regulation of the promoter and terminator regions of *zpg* show high rates of biased transmission and substantially improved fertility compared with the *vasa*2 promoter at a previously validated female fertility locus (*AGAP007280*)

Phenotypic assays were performed to measure fertility and transmission rates for each gene drive based upon the *vasa* and *zpg* promoters. The data for the *vasa-CRISPR*^{h} is previously reported in Hammond et al. (2016). The *zpg-CRISPR*^{h} construct targeting AGAP007280 recognised exactly the same target site and was inserted in identical fashion to the *vasa-CRISPR*^{h}, through recombinase-mediated cassette exchange[9]. The larval output was determined for individual drive heterozygotes crossed to wild type (left), and their progeny scored for the presence of DsRed linked to the construct (right). The average progeny count and transmission rate is also shown (± s.e.m.).

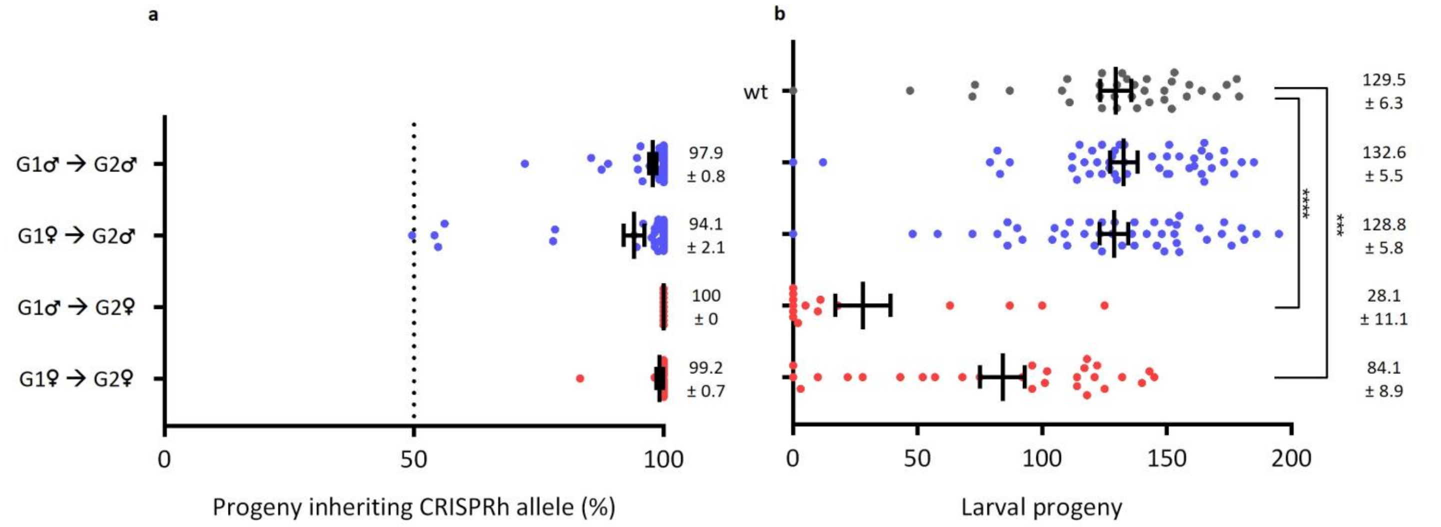

Supplementary Figure 5

Maternal or paternal inheritance of the *dsxF^{CRISPRh}* driving allele affect fecundity and transmission bias in heterozygotes

Male and female *dsxF^{CRISPRh}* heterozygotes (*dsxF^{CRISPRh}/+*) that had inherited a maternal or paternal copy of the driving allele were crossed to wild type and assessed for inheritance bias of the construct (**a**) and reproductive phenotype (**b**). (**a**) Progeny from single crosses (n≥15) were screened for the fraction that inherited DsRed marker gene linked to the *dsxF^{CRISPRh}* driving allele (e.g. G_1♂→G_2♀ represents a heterozygous female that received the drive allele from her father). Levels of homing were similarly high in males and females whether the allele had been inherited maternally or paternally. The dotted line indicates the expected Mendelian inheritance. Mean transmission rate (± s.e.m.) is shown. (**b**) Counts of hatched larvae for the individual crosses revealed a fertility cost in female *dsxF^{CRISPRh}* heterozygotes that was stronger when the allele was inherited paternally. Mean progeny count (± s.e.m.) is shown. (***, p<0.001;****, p<0.0001; Kruskal-Wallis test).

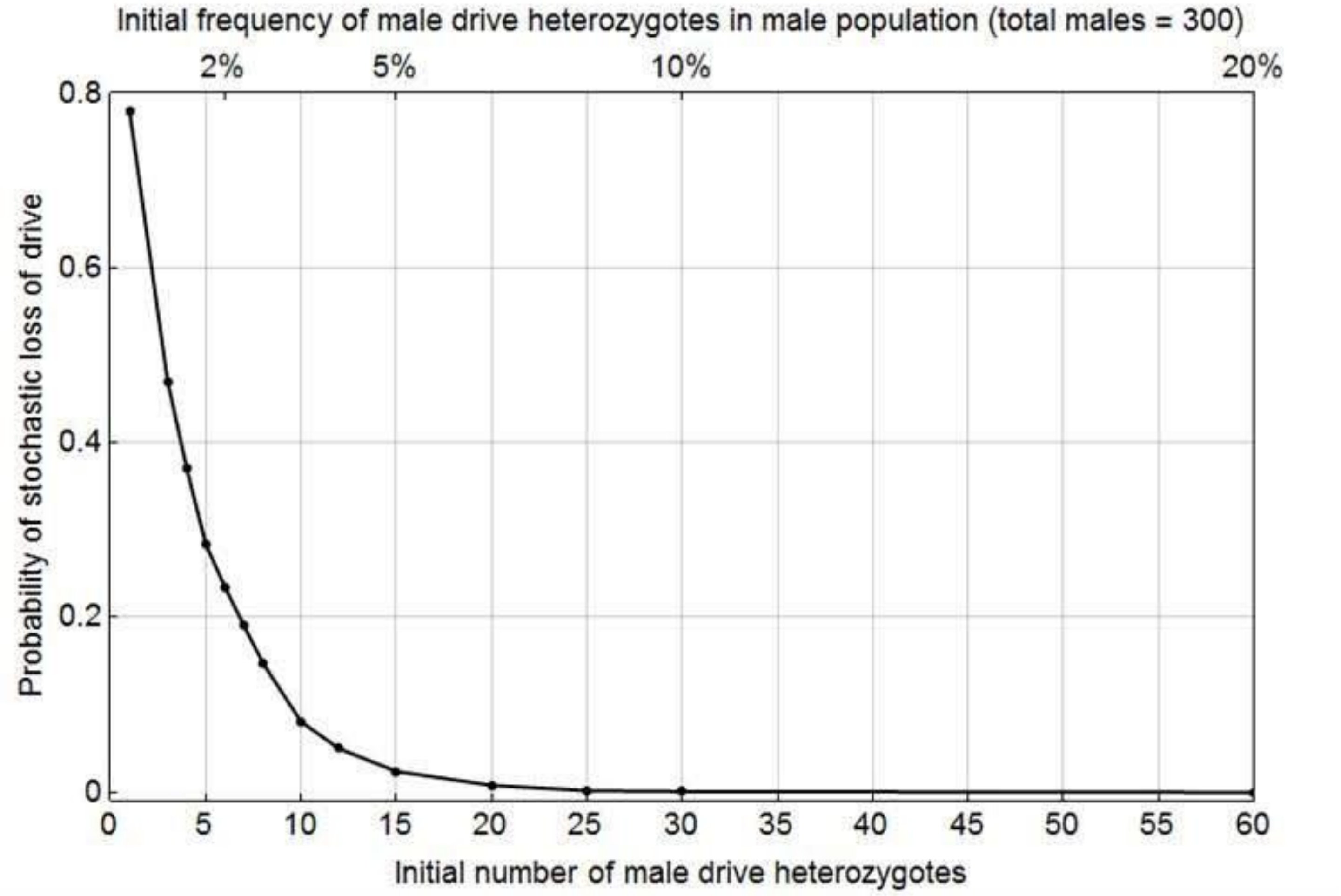

Supplementary Figure 6

Probability of stochastic loss of the drive as a function of initial number of male drive heterozygotes

To calculate the probability of stochastic loss of the drive in the cage experiment setup, for each initial number (h0) of male drive heterozygous individuals, out of 1000 simulations of the stochastic cage model (described in Supp Info), we recorded the number of times the drive was not present at 40 generations (and consequently population elimination did not occur). Each data point represents 1000 individual simulations of the stochastic cage model.

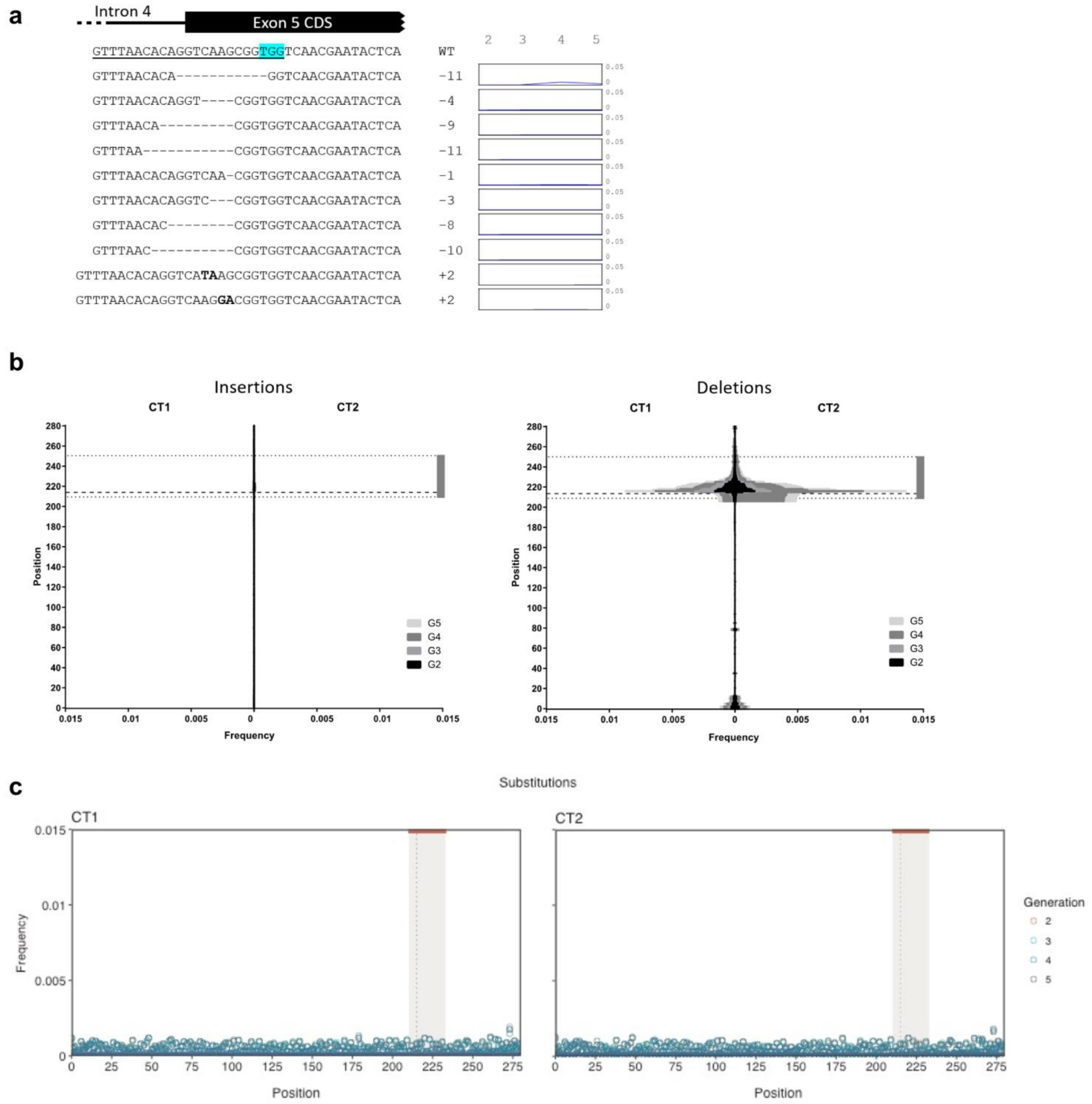

Supplementary Figure 7

Frequency plots of variants and indels in target sequence

Pooled amplicon sequencing of the target site from 4 generations of the cage experiment (generations 2, 3, 4 and 5) revealed a range of very low frequency indels at the target site (**a**), none of which showed any sign of positive selection. Insertion, deletion and substitution frequencies per nucleotide position were calculated, as a fraction of all non-drive

alleles, from the deep sequencing analysis for both cages. Distribution of insertions and deletions (**b**) in the amplicon is shown for each cage. Contribution of insertions and deletions arising from different generations is displayed with the frequency in each generation represented by a different colour. Significant change ($p<0.01$) in the overall indel frequency was observed in the region around the cut-site (dotted area ± 20 bp) for both cages. No significant changes were observed in the substitution frequency (**c**) around the cut-site (shaded area ± 20 bp) when compared with the rest of the amplicon, confirming that the gene drive did not generate any substitution activity at the target locus and that the laboratory colony is devoid of any standing variation in the form of SNPs within the entire amplicon.

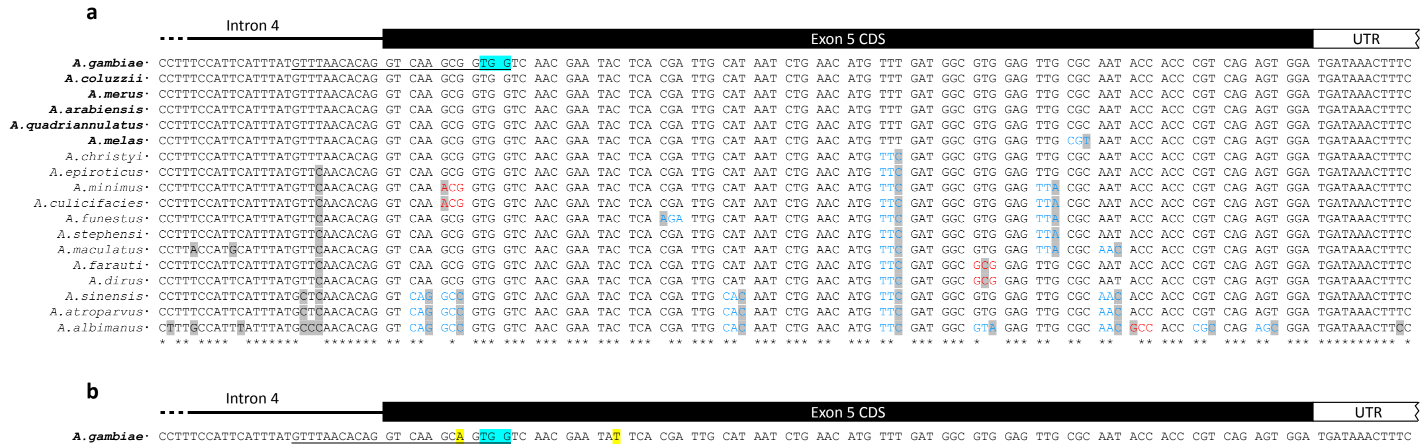

Supplementary Figure 8

Sequence comparison of the *dsx* female-specific exon 5 across members of the *Anopheles* genus and SNP data obtained from *A. gambiae* mosquitoes in Africa.

(**a**) Sequence comparison of the *dsx* intron 4-exon 5 boundary and the *dsx* female-specific exon 5 within the 16 anopheline species[16]. The sequence of the intron 4-exon 5 boundary is completely conserved within the six species that form the *Anopheles gambiae* species complex (noted in **bold**). The gRNA used to target the gene is underlined and the PAM is highlighted in blue. Changes in the DNA sequence are shaded grey and codon silent and missense substitutions are noted in blue and red respectively. (**b**) SNP frequencies obtained from 765 *Anopheles gambiae* mosquitoes captured across Africa[17]. Across the *dsx* female-specific Exon 5 there are only 2 SNP variants (noted in yellow) with frequencies of 2.9% (the SNP in the gRNA-complementary sequence) and 0.07%.

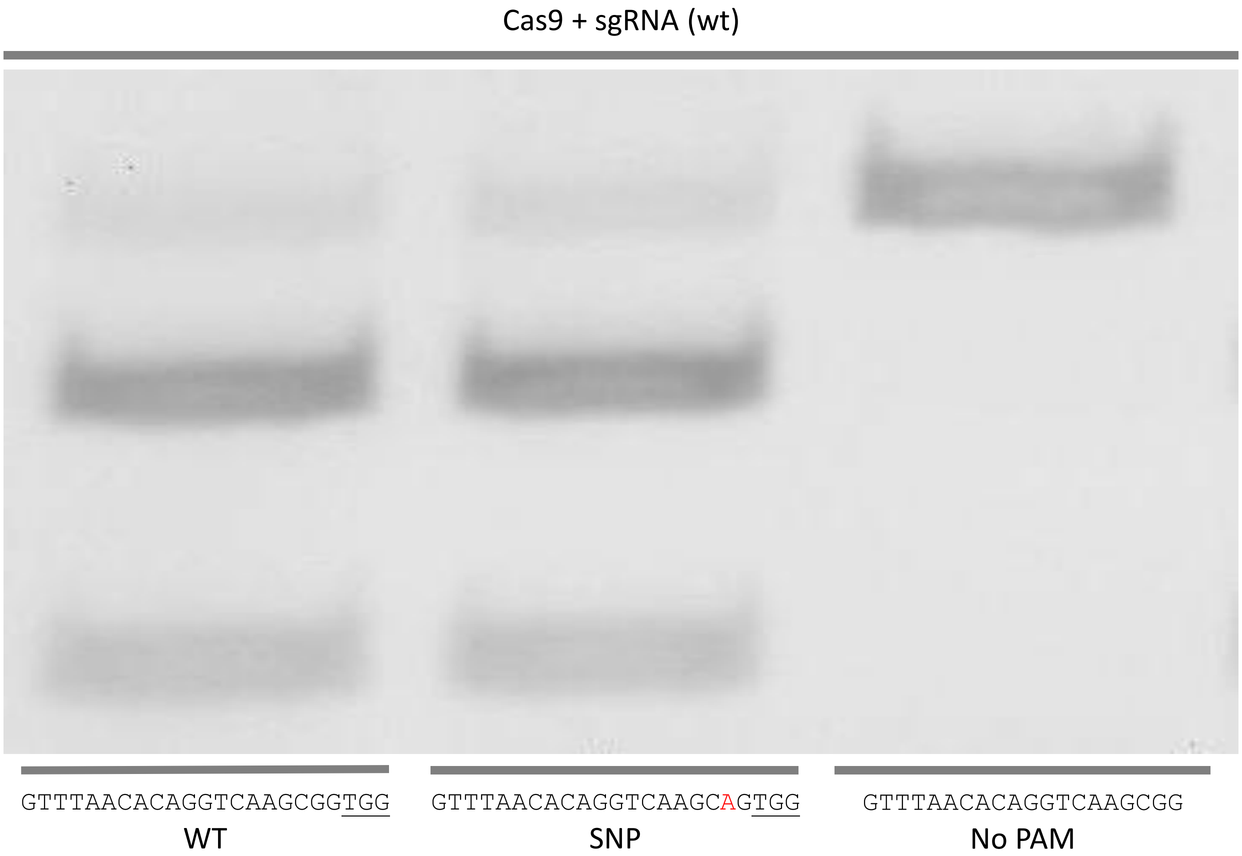

Supplementary Figure 9

***In vitro* cleavage assay testing the efficiency of the gRNA in the *dsxF*^{CRISPRh} gene drive to cleave the *dsx* exon 5 target site with the SNP found in wild populations in Africa**

An in vitro cleavage assay using an RNP complex of Cas9 enzyme and the gRNA used in this study was performed against linearised plasmids containing either wild-type (WT) target site in *dsx* exon 5 or the same site containing the single SNP found in wild caught populations (SNP). Products of the in vitro cleavage assay were purified and analysed on a gel. Both the WT and SNP-containing target sites are susceptible to the cleavage activity of the RNP complex as shown by the diminished high molecular band and the presence of the two cleavage products of the expected size. A *dsx* exon 5 target site containing the WT sequence complementary to the gRNA but without the PAM sequence was used as a control ('no PAM').

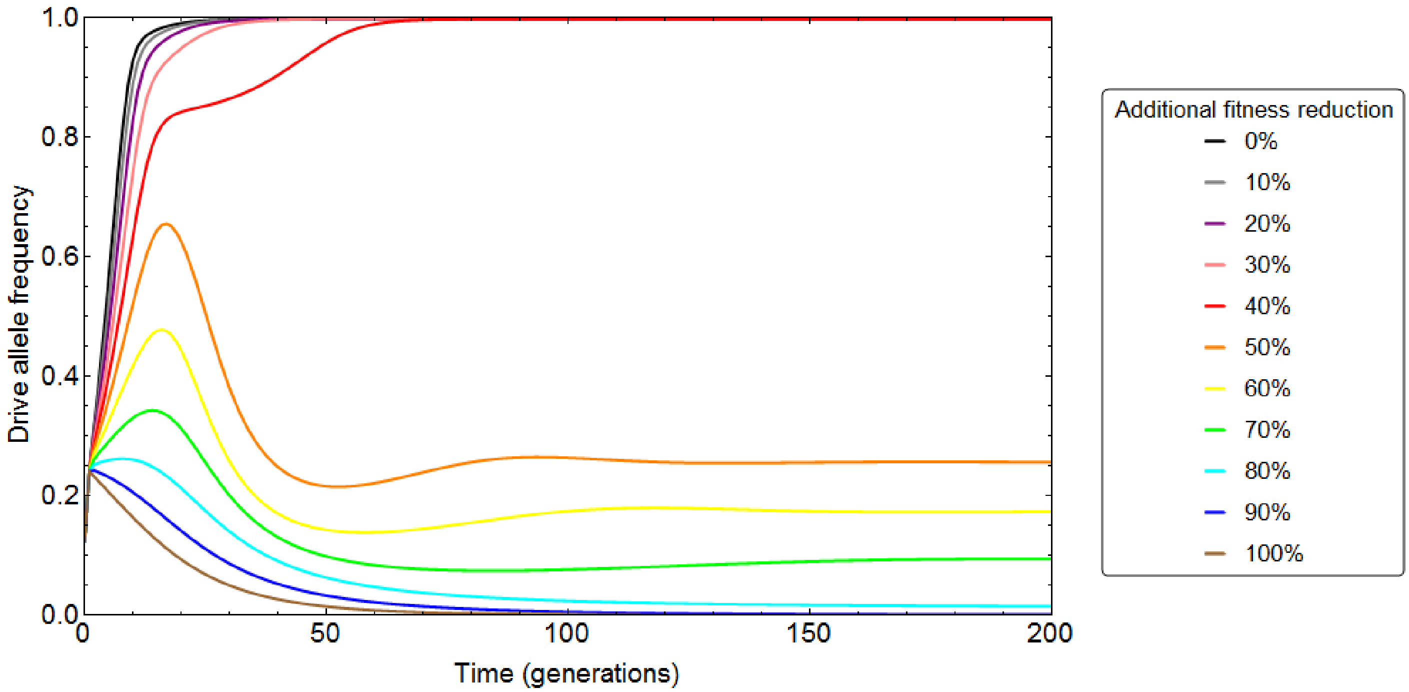

Supplementary Figure 10

Modeling the effect of unforeseen additional fitness reduction encountered by heterozygous gene drive females

Time dynamics of drive allele frequency as predicted by the deterministic model, with different coloured lines representing additional percentage reductions from zero to 100% in the baseline fertility of females, mimicking an ecologically more realistic scenario in which there were more severe fitness effects associated with the gene drive than in the laboratory. The reduction in fitness is assumed to affect the overall reproductive success (i.e mating success, longevity, fertility etc.). The baseline fertility values relative to the wild type are those reported in Supplementary Table S4 (0.65 for females with transgenic mothers, and 0.217 for females with transgenic fathers), that describes these and all other parameters estimated from experiment. Fitness reductions of up to 40% are predicted to crash the population. The spread of the drive is computed using the deterministic model in Figure 5.
