# Supplementary material for: A CRISPR–Cas9 gene drive targeting doublesex causes complete population suppression in caged Anopheles gambiae mosquitoes
Source: Nat Biotechnol. 2018 Sep 24;36(11):1062–6. doi: 10.1038/nbt.4245 (PMC6871539; doi:10.1038/nbt.4245)
Supplement: Supplementary Tables — 1–6 (PDF 1046 kb) [file 41587_2018_BFnbt4245_MOESM38_ESM.pdf]

SUPPLEMENTARY TABLES

Supp Table S1 Comparison of performance of gene drive and conventional genetic control approaches in terms of fitness and generation of resistance

Insect Species	Genetic modification	Intended control measure	Field/semi-field fitness	Homing rate	Resistance rate	Selection of resistance in cage	Population suppression	Study
Drosophila melanogaster	*tra^{nCHE}* targeting the *transformer* gene	Gene Drive	Not tested	56% males<br>0% females	Male germline: 28% r2, 14% r1	yes	no	KaramiNejadRanjbar et al. 2018
Drosophila melanogaster	*D-white(2-gRNA)* targeting the X-linked *white* gene, two separate gRNAs	Gene Drive	Not tested	76% males<br>0% females	Male germline: 23% r2, 0% r1; Embryonic 77% r2,0% r1	Not tested	Not tested (not designed for suppression)	Champer et al. 2018
Drosophila melanogaster	*D-cinnabar* targeting the *cinnabar* gene	Gene Drive	Not tested	38% males;<br>54% females	Male germline: 62% r2, 0% r1<br>Female germline: 46% r2, 0%r1<br>Embryonic: 100% r2	Not tested	Not tested (not designed for suppression)	Champer et al. 2018
Anopheles stephensi	*AsMCRkh2* targeting the *kynurenine hydroxylase* carrying a single chain antibody	Gene Drive	Not tested	97% males<br>99%females	Germline: not determined; embryonic: detected due to maternal deposition	Not tested	Not tested (not designed for suppression)	Gantz et al. 2015

Nature Biotechnology: doi:10.1038/nbt.4245

Anopheles gambiae	*vasa-CRISPR*[h] targeting the autosomal gene *nudel* (*AGAP007280*)	Gene Drive	Not tested	99% males<br>95% females	males:<br>0.28% r2, 0.14% r1<br>embryonic due to maternal deposition: 80%	yes	no	Hammond et al. 2016; Hammond et al. 2017
Anopheles gambiae	*zpg-CRISPR*[h] targeting female *dsx* exon 5 (*AGAP004050*)	Gene Drive	Not tested	92% males<br>99% females	*male:<br>4.6% r2, 0% r1	no	yes	This study
Aedes aegypti	OX513A, a construct causing dominant lethality	RIDL SIT	0.56	n.a	n.a	n.a	n.a	AF Harris et al. 2011
Aedes aegypti	OX3604C a construct causing a female-specific flightless phenotype	RIDL SIT	0.03	n.a	n.a	n.a	n.a	Facchinelli et al. 2013

*Among the rare offspring of males that did not contain the drive allele (8 %) we sequenced 27 individuals, 12 of which had the wild-type allele and 15 of which had a putative non-functional resistant (r2) allele - either an out of frame 11bp deletion consistent with microhomology-mediated end joining, or a partial homing event.

**Supp Table S2 | Ratio of larvae recovered by intercrossing heterozygous *dsx* ϕC31-knock-in mosquitoes**			
**GFP strong (*dsxF$^{-/-}$*)**	**GFP weak (*dsxF$^{-/+}$*)**	**no GFP (*+/+)***	**Total**
**262 (24.9%)**	**523 (49.7%)**	**268 (25.5%)**	**1053**

Supp Table S2 | Heterozygous and homozygous individuals for the *dsxF^{-}* allele were separated based on the intensity of fluorescence afforded by the GFP transcription unit within the knockout allele. Homozygous mutants were distinguishable as recovered in the expected Mendelian ratio of 1:2:1 suggesting that the disruption of the female-specific isoform of *Agdsx* is not lethal at the L1 larval stage.

**Supp Table S3 | Genetic females homozygous for the insertion carry male-specific characteristics**						
	**Genetic Males**			**Genetic Females**		
Characteristic	***dsxF*** $^{+/+}$	***dsxF*** $^{+/-}$	***dsxF*** $^{-/-}$	***dsxF*** $^{+/+}$	***dsxF*** $^{+/-}$	***dsxF*** $^{-/-}$
Pupal genital lobe	male	male	male	female	female	male
Claspers	✓	✓	✓	✗	✗	✓
Cercus	✗	✗	✗	✓	✓	✗
Spermatheca	✗	✗	✗	✓	✓	✗
MAGs	✓	✓	✓	✗	✗	✓
Feed on blood	✗	✗	✗	✓	✓	✗
Can lay eggs	✗	✗	✗	✓	✓	✗
Plumose antennae	✓	✓	✓	✗	✗	✓
Pilose antennae	✗	✗	✗	✓	✓	✗

Supp Table S3 Phenotypic characteristics observed on adult mosquitoes taken from the *dsxF* $^{-}$ crosses. Female mosquitoes of the *dsxF* $^{-/-}$ class present a profile of characteristics that matches the male sex rather than the female.

Supp Table S4 | Primers used in this study	
dsxgRNA-F	TGCTGTTTAACACAGGTCAAGCGG
dsxgRNA-R	AAACCCGCTTGACCTGTGTTAAAC
dsxϕ31L-F	GCTCGAATTAACCATTGTGGACCGGTCTTGTGTTTAGCAGGCAGGGGA
dsxϕ31L-R	CACCAAGACAGTTAACGTATCCGTTACCTTGACCTGTGTTAAACATAAAT
dsxϕ31R-F	GGTGGTAGTGCCACACAGAGAGCTTCGCGGTGGTCAACGAATACTCACG
dsxϕ31R-R	TCCACCTCACCCATGGGACCCACGCGTGGTGCGGGTCACCGAGATGTTC
zpgprCRISPR-F	GCTCGAATTAACCATTGTGGACCGGTCAGCGCTGGCGGTGGGGA
zpgprCRISPR-R	TCGTGGTCCTTATAGTCCATCTCGAGCTCGATGCTGTATTTGTTGT
zpgteCRISPR-F	AGGCAAAAAAGAAAAAGTAATTAATTAAGAGGACGGCGAGAAGTAATCAT
zpgteCRISPR-R	TTCAAGCGCACGCATACAAAGGCGCGCCTCGCATAATGAACGAACCAAAGG
dsxin3-F	GGCCCTTCAACCCGAAGAAT
GFP-F	GCCCTGAGCAAAGACCCCAA
dsxex4-F	GCACACCAGCGGATCGACGAAG
dsxex5-R	CCCACATACAAAGATACGGACAG
dsxex6-R	GAATTTGGTGTCAAGGTTCAGG
3xP3	TATACTCCGGCGGTCGAGGGTT
hCas9-F	CCAAGAGAGTGATCCTGGCCGA
dsxex5-R1	CTTATCGGCATCAGTTGCGCAC

Nature Biotechnology: doi:10.1038/nbt.4245

dsxin4-F	GGTGTTATGCCACGTTCACTGA
RFP-R	CAAGTGGGAGCGCGTGATGAAC
† Dsx-original-target-F	TAGGGTTTAACACAGGTCAAGCGGTGG
† Dsx-original-target-R	AAACCCACCGCTTGACCTGTGTTAAAC
† Dsx-SNP-target-F	TAGGGTTTAACACAGGTCAAGCAGTGG
† Dsx-SNP-target-R	AAACCCACTGCTTGACCTGTGTTAAAC
† Dsx-noPAM-target-F	TAGGTTTAACACAGGTCAAGCGG
† Dsx-noPAM-target-R	AAACCCGCTTGACCTGTGTTAAA

Supp Table S4 Table listing the primers used in this study. Gibson assembly and Golden Gate cloning overhangs are underlined with a single and a double line respectively. † Primers used to create the target sequences for the *in vitro* RNP cleavage assay.

Supp Table S5 | Parameters for stochastic cage model		
**Parameter**	**Estimate**	**Method of estimation**
Mating probability	0.85 for heterozygotes; 0 for D/D, D/R and R/R homozygotes	Estimated from Hammond et al. 2017
Egg production from wild-type female (no parental nuclease)	Mean 137.4. Sampling with replacement of observed values (10, 61, 96, 98, 111, 111, 113, 127, 128, 129, 132, 132, 134, 135, 137, 138, 138, 139, 142, 142, 146, 146, 149, 152, 152, 152, 158, 160, 162, 164, 170, 179, 186, 189, 191)	From assays of mated females
Egg production from W/D heterozygote female (nuclease from ♀)	Mean 118.96. Sampling with replacement of observed values (12, 31, 76, 90, 96, 100, 106, 106, 107, 113, 117, 118, 119, 130, 133, 136, 136, 136, 137, 138, 139, 142, 143, 145, 146, 148, 157, 174)	From assays of mated females
Egg production from W/D heterozygote female (nuclease from ♂)	Mean 59.67. Sampling with replacement of observed values (0, 0, 0, 0, 0, 34, 47, 50, 65, 105, 113, 115, 115, 125, 126)	From assays of mated females
Hatching probability, wild-type female (no parental nuclease)	0.941	From assays of mated females
Hatching probability, W/D heterozygote female (nuclease from ♀)	0.707	From assays of mated females
Hatching probability,	0.47	From assays of mated females

W/D heterozygote female (nuclease from ♂)		
Probability of emergence from pupa (survival from larva)	0.8708	Average of observations over all generations and both cage experiments
Drive in W/D females	0.9985	Observed fraction transgenic from assays
Drive in W/D males	0.9635	Observed fraction transgenic from assays
Meiotic EJ parameter (fraction non-drive alleles that are resistant)	0.4685	Estimated from Hammond et al. 2016

Supp Table S5 **|** Parameters for stochastic cage model

We assume that parental effects on fitness (egg production and hatching rates) for non-drive (W/W, W/R) females with nuclease from one or both parents are the same as observed values for drive heterozygote (W/D) females with parental effects. For combined maternal and paternal effects (nuclease from both parents), the minimum of the observed values for maternal and paternal effect is assumed.

Supp Table S6 | Summary of values obtained from the cage trials								
	Cage Trial 1				Cage Trial 2			
Generation	**Transgenic Rate (%)**	**Hatching Rate (%)**	**Egg Output (N)**	**Repr. Load (%)**	**Transgenic Rate (%)**	**Hatching Rate (%)**	**Egg Output (N)**	**Repr. Load (%)**
G0	25 (150/600)	-	27462	-	25 (150/600)	-	26895	-
G1	49.65 (286/576)	88.62 (576/650)	17405	36.62	50 (280/560)	86.15 (560/650)	16578	38.36
G2	62.01 (302/487)	74.92 (487/650)	14957	45.54	61.79 (325/526)	80.92 (526/650)	15565	42.13
G3	68.94 (344/499)	76.77 (499/650)	11249	59.04	68.05 (328/482)	74.15 (482/650)	9376	65.14
G4	67.67 (316/467)	71.85 (467/650)	9170	66.61	85.41 (398/466)	71.69 (466/650)	6514	75.78
G5	58.67 (264/450)	69.23 (450/650)	11364	58.62	86.5 (346/400)	61.54 (400/650)	4805	82.13
G6	63.3 (288/455)	70 (455/650)	7727	71.86	90.09 (309/343)	52.77 (343/650)	4210	84.35
G7	69.47 (355/511)	78.62 (511/650)	7785	71.65	100 (363/363)	55.85 (363/650)	1668	93.8
G8	70.07 (323/461)	70.92 (461/650)	6293	77.08	100 (278/278)	42.77 (278/650)	0	100
G9	75.58 (325/430)	66.15 (430/650)	4107	85.04	-	-	-	-
G10	95.71 (357/373)	57.38 (373/650)	4146	84.90				
G11	100 (374/374)	57.54 (374/650)	2645	90.37				
G12	100 (253/253)	38.92 (253/650)	0	100				

Supp Table S6 | Summary of values obtained from the cage trials

Transgenic rate, hatching rate, egg output and reproductive load at each generation during the cage experiment. The reproductive load indicates the suppression of egg production at each generation compared to the first generation.
